# Switchable High-Valent Ag^3+^/Ag^+^ Redox Pair Stabilized in Polyoxometalate as Highly Oxidative “Electron Shuttle” Catalysts

**DOI:** 10.1021/jacsau.5c00987

**Published:** 2025-10-14

**Authors:** Xiang Li, Hehua Hui, Yuanhang Ren, Zhaoqing Liu, Yiyang Li, Bin Yue, Heyong He, Shik Chi Edman Tsang

**Affiliations:** † Department of Chemistry, 6396 University of Oxford, Oxford OX1 3QR, England, U.K.; ‡ Department of Chemistry and Shanghai Key Laboratory of Molecular Catalysis and Innovative Materials, 12478 Fudan University, Shanghai 200438, China; § College of Chemistry and Molecular Engineering, 12465 Peking University, Beijing 100871, China

**Keywords:** polyoxometalates, silver, electrochemistry, oxidation, redox chemistry, electrocatalysis

## Abstract

We report the isolation and characterization of the first example of a crystallized high-valent Ag^3+^-containing polyoxometalate (POM) complex, Cs_7_K_4_[P_2_W_19_Ag^III^O_69_(OH_2_)]·17H_2_O (**1**), as a “catalyst bearing catalyst” capable of catalyzing the formation of the high-valent Ni^3+^-containing POMs in the presence of peroxydisulfate. The oxidation state and exotic chemical behaviors of Ag in **1** were confirmed by crystallographic, spectroscopic, and electrochemical characterizations. The Ag^3+^/Ag^+^ redox pair embedded in **1** showed good electrochemical reversibility and the ability to accelerate electron transfer, contributing to the observed catalytic activity of **1** in both formation of other high-valent metal-containing POMs and electrochemical oxidative C–H activation.

## Graphical abstract

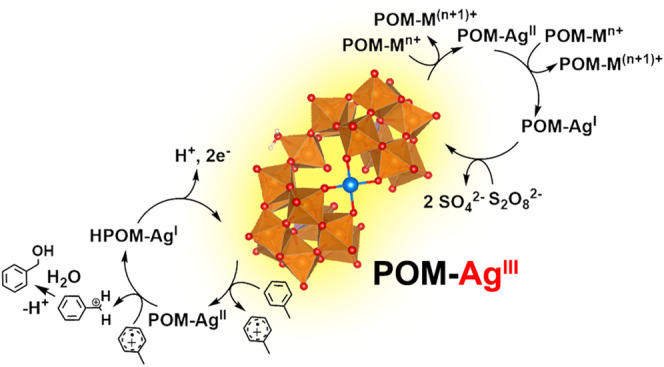

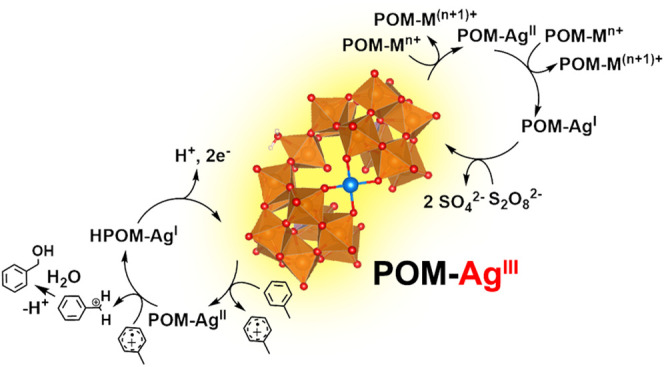

## Introduction

Oxidative high-valent transition-metal centers have been confirmed to be active catalytic intermediates in various redox reactions.^1,2^ Occurrences of oxidative species containing Fe^4+^ and Cu^3+^ are either observed or proposed for various enzymatic redox reactions, and the existence of high-valent metal centers has also been extensively studied in different systems.^3–6^ Specifically, the stability and reactivity of these high-valent species are considered crucial to many catalytic oxidation reactions such as electrochemical water oxidation.^7^ A large proportion of such catalytic compounds undergo unwanted side reactions and decompositions of the species due to either significant structural changes driven by the redox process of the metal centers or the oxidative degradation of organic ligands used to stabilize the metal centers (in the case of ligand-stabilized complexes), leading to lower reversibility.^8–11^ A noticeable example of such transition-metal centers would be silver. Previous studies have suggested its ability to catalyze oxidation reactions with promising efficiency,^11–14^ but the oxidation of Ag^+^ to higher valent Ag^2+^/Ag^3+^ could result in dramatic coordination structural changes of the Ag centers during redox reactions, e.g., from the linear geometry of Ag^+^ to the square planar coordination geometry of Ag^2+^/^3+^, as a result of different crystal field stabilization energies (CFSEs) due to their different electron configurations (d^10^ for Ag^+^, d^9^ for Ag^2+^, and d^8^ for Ag^3+^). The structure of the whole complex may change accordingly, resulting in decomposition of the complex. Hence, the structural reversibility between high- and low-valent silver species is affected, further undermining their stability and catalytic efficiency.

Meanwhile, polyoxometalates (POMs), a series of catalytically active metal–oxygen clusters with the ability to transfer multiple electrons in redox reactions, could be potential all-inorganic ligands that provide a promising solution to both structural change and ligand stability issues. The large, rigid structures of the POMs as whole clusters can remain stable in the redox process, while their resistance toward oxidation conditions prevents the oxidative degradation of these stabilizing ligands.^15–17^ Silver-based POMs with high redox potentials are well-known to be capable of catalyzing a number of important chemical reactions.^18–21^ Notably, the presence of strongly oxidative high-valent Ag^3+^ intermediates has been proposed for the catalytic oxidation of water, but no evidence is yet there on the oxidation state of Ag and the structural proof of these intermediates.^21^ High-valent silver intermediates are also postulated to play an important role in our developed silver-assisted peroxydisulfate oxidation method in producing other high-valent transition metals, such as Cu^3+^- and Ni^3+^-containing POM complexes, and these complexes cannot be obtained without the presence of silver-containing catalysts.^16,22^ Importantly, a direct confirmation of the high-valent Ag^3+^ in POMs is required for further in-depth studies, which could help to expand the palette of Ag-based POM materials and develop new Ag-based redox catalysts.

Herein, we report a crystallized POM containing oxidative high-valent Ag^3+^, Cs_7_K_4_[P_2_W_19_Ag^III^O_69_(OH_2_)]·17H_2_O (**1**). The crystallized compound confirmed the successful stabilization of the high-valent Ag centers, which exhibit interesting properties regarding its structural features and reactivities. Specifically, the electrochemical reversibility and the strong oxidation ability observed in this study revealed its intriguing redox properties as an “electron shuttle” that accelerates the electron-transfer process, showing its potential as a strongly oxidative electrochemical redox catalyst.

## Results and Discussion

Compound **1** is separated from the reaction mixture during the synthesis of a high-valent Ni^3+^-containing POM, Cs_4_K­[PW_9_Ni^III^_2_Ni^II^O_40_H_8_]·6H_2_O,^6^ which indicates that compound **1** functions as the active high-valent silver intermediate to oxidize Ni^2+^. Using a modified procedure of increasing the amount of [PW_9_O_34_]^9–^ and Ag^+^ contents in the reactant mixture, dark-orange crystals of **1** can be obtained as the only crystalline product (Figure S1) along with large amounts of amorphous Cs_4_K­[PW_9_Ni^III^_2_Ni^II^O_40_H_8_]·6H_2_O. The preparation can be well repeated with a rather low but stable yield; interestingly, despite not occurring in the final product, the presence of Ni^2+^ seemed to be required for the formation of **1**. We postulate that the anion [PW_9_Ni^III^_2_Ni^II^O_40_H_8_]^5–^ formed in the reaction might play the role of buffering agents, which stabilizes the pH value of the reaction mixture to ca. 7, allowing the successful formation and separation of **1**. Elemental analysis and thermogravimetric results are also in good agreement with the formula obtained from the crystal structure (Figure S2). Solid **1** is stable for up to 2 months under 0 °C without significant decomposition but slowly fades, followed by the formation of a reddish-brown mixture of insoluble decomposition products during storage. The compound **1** is slightly soluble in water, giving a yellow solution with a pH value of 7–8, which completely fades within 2 days (see below).

Compound **1** crystallizes as a monoclinic crystal in the space group *C2/c* with the lattice constants *a* = 20.53 Å, *b* = 16.09 Å, *c* = 26.63 Å, and β = 102.45° (see Table S1, Figures 1a and S3). The clearly distinguishable sandwich-like anion [P_2_W_19_Ag^III^O_70_H_2_]^11–^ (see Figure 1a,b) has two A-α-[PW_9_O_34_]^9–^ ligands coordinated to two 50% occupied WO­(OH_2_)^4+^ units, originates from the crystallographic disorder of the anions, and represents the same WO­(OH_2_)^4+^ moiety on the central horizontal “belt” along with a partially occupied (63%) Ag site (represented as Ag1 below) with the square planar coordination geometry characteristic for the d^8^ transition-metal ion. For a given Ag ion size, the short average Ag–O bond length of 2.04 Å (Figure 1c) indicative of a strong Ag^3+^-O interaction fits well with the reported value for Ag^3+^-O bonds.^23^ BVS calculation result of the Ag1 center^24^ revealed its oxidation state as +3. This bond length as well as the diagonal O–Ag–O bond angle (170°) are notably larger than the corresponding values of WO­(OH_2_)^4+^ units in **1** (average W–O bond length on the plane 1.96 Å; diagonal O–W–O bond angle 162°) and other similar structures,^25^ satisfactorily distinguishing Ag1 as an Ag^3+^ site from a disordered WO­(OH_2_)^4+^ site, a disorder phenomenon observed in some previously reported sandwich-type POMs.^25^ To the best of our knowledge, compound **1** represents an exotic example of a confirmed Ag^3+^-containing POM. Another partially occupied (37%) Ag site, namely, Ag2, occurs noticeably close to Ag1 (see additional notes in the Electronic Supporting Information). Ag2 has a coordination number of 5 and Ag–O bond lengths varying between 2.47 and 2.59 Å (Figure 1d) which is in line with Ag^+^ centers as further confirmed by BVS calculations (Table S2).^24^ The total occupancy of the two Ag sites as 1.0 even when Ag sites were separately refined indicates that Ag2 is likely formed by the in situ reduction of the high-valent Ag1 centers in the crystal by water molecules during storage, while the overall structure of the POM framework remains unchanged. Upon prolonged storage, Figure S4a shows that the P–O vibration peak is gradually reduced from 1083 to 1071 cm^–1^, along with the shift of the terminal W–O vibration peak from 948 to 940 cm^–1^ and strengthening of the 890 cm^–1^ peak, while the general features of the IR spectra, e.g., the number and approximate position of peaks, remain unchanged, indicating that the overall structure of POMs does not change. The retention of the overall POM structure is also confirmed by the Raman spectra recorded before and after reduction of the samples (Figure S4b). Thus, the change in the IR spectra does not include structural rearrangements or decomposition and can only be attributed to the substituent metal. The effect of such metal centers on the P–O stretching peak, especially regarding their changes in oxidation states, has been well-explored in previous studies.^16,22^ In the case of **1**, it is expected that the reduction of Ag^3+^ to Ag^+^ leads to the weakening of electrostatic forces between Ag centers and the POM frameworks, and the anion structure relaxes from the strongly bound Ag^3+^-O in [P_2_W_19_Ag^III^O_70_H_2_]^11–^, resulting in an IR spectrum closer to pristine [P_2_W_19_O_69_(OH_2_)]^14–^.^25^ This result further confirmed the successful introduction of Ag^3+^ centers into the POM framework, as such time-dependent variations cannot be explained by structural models excluding the effect of the spontaneously reducing substituent Ag^3+^. Meanwhile, the possible existence of the paramagnetic Ag^2+^ in both crystals and a solution of **1** seemed highly unlikely, as revealed by the silent EPR measurement results (Figure S5).

**Figure 1:**
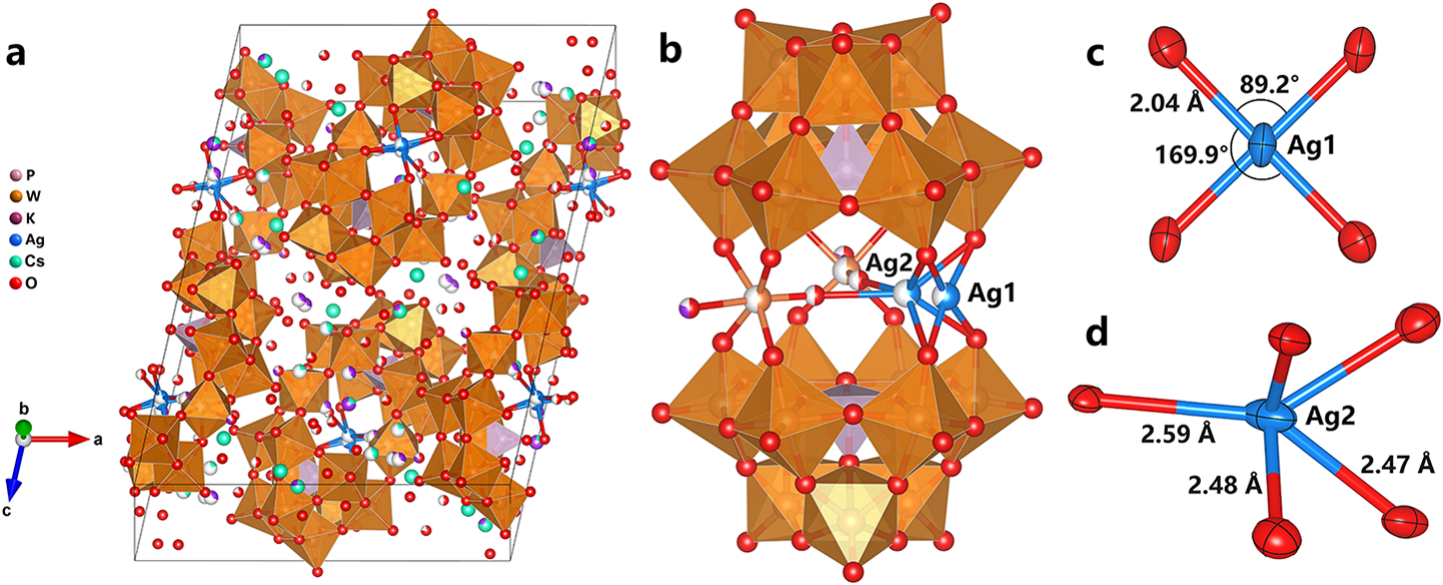
Crystal structure of **1** (a), structure of the POM anions showing partially occupied Ag, W, and O sites (b), and detailed coordination geometry of Ag1 (c) and Ag2 (d).

We further conducted computational studies on a series of related anions with three different oxidation states of Ag including [P_2_W_19_Ag^III^O_70_H_2_]^11–^, [P_2_W_19_Ag^II^O_70_H_2_]^12–^, and [P_2_W_19_Ag^I^O_70_H_2_]^13–^ (see Figure 2a–c and Tables S3–S5).^26–32^ In the case of [P_2_W_19_Ag^I^O_70_H_2_]^13–^, Ag^+^ adopted a distorted pyramidal coordination configuration closely similar to the observed coordination geometry of the Ag2 site in the structure as discussed above. The Ag–O bond length varies in the range of 2.41–2.88 Å, suggesting a rather flexible electrostatic interaction between the univalent Ag^+^ and the negatively charged POM framework. Meanwhile, the optimized structure of [P_2_W_19_Ag^III^O_70_H_2_]^11–^ confirmed the square planar coordination geometry of Ag^3+^ with an average Ag^3+^-O bond length of 1.99 Å and a diagonal Å–Ag-O bond angle of 173°. In comparison, the optimized average planar W–O bond length and the diagonal O–W–O bond angle are 1.91 Å and 156°, respectively. These results are quite close to the refined geometric parameters for Ag1 and WO­(OH_2_)^4+^ sites and confirm their differences in geometric features, well supporting that Ag1 is a POM-embedded trivalent silver center. The oxidation state of Ag in [P_2_W_19_Ag^III^O_70_H_2_]^11–^ as +3 is also confirmed by the localized orbital bonding analysis method,^33,34^ while all oxygen atoms have an oxidation state closer to −2, indicating that [P_2_W_19_Ag^III^O_70_H_2_]^11–^ is a real Ag^3+^ complex from the perspective of its electronic structure.^35^ The same coordination geometry is also observed for the Ag^2+^ center in the postulated [P_2_W_19_Ag^II^O_70_H_2_]^12–^ but with a longer Ag^II^–O bond length varying between 2.11 and 2.17 Å with an average value of 2.13 Å. It is interesting to confirm that the overall POM framework remains the same regardless of the oxidation states of Ag centers, in line with the crystallographic result. The electron localization function (ELF) analysis^36^ (see Figure 2d–f) on [P_2_W_19_Ag^III^O_70_H_2_]^11–^ revealed a primarily ionic character of the Ag–O bond, suggesting that the stabilization of the high-valent Ag is dominated by the electrostatic forces between Ag^3+^ and O^2–^, while the high charge of Ag^3+^ results in a stronger interaction as observed in the crystallographic results. Given the electrostatic nature of the Ag–O interaction, the coordination structure of Ag^3+^ can accordingly be explained by the crystal field theory (CFT), where the d-orbital splitting of the square-planar-coordinated Ag^3+^ results in a large CFSE that dominates its coordination geometry. Molecular orbital (MO) analyses revealed the Ag^3+^ 4d orbital components in [P_2_W_19_Ag^III^O_70_H_2_]^11–^ being widely distributed energetically over a number of low-lying MOs (Figure S6a) except the lowest unoccupied molecular orbital that the Ag^3+^ 4*d*_*x* ^2^–*y* ^2^_ orbital mostly contributes to (Figure S6b), which lies much higher than other Ag 4d-related MOs, corresponding to the CFT-predicted d-orbital splitting in a square planar crystal field. The highest occupied molecular orbital (HOMO) is mainly composed of oxygen 2p orbitals. In contrast, the HOMO of [P_2_W_19_Ag^I^O_70_H_2_]^13–^ is mainly the Ag^+^ 4*d*_*x* ^2^–*y* ^2^_ orbital, while other highest occupied MOs also show significant Ag 4d character (Figure S7), all of which are close in energy. In both cases, the higher unoccupied MOs are mainly composed of W 6d orbitals (Figures S6b and S7b). Meanwhile, it is also worth mentioning that even more distant oxygen atoms within the highly negative-charged oxygen cavity are anticipated to play a role in anchoring Ag^+^ in [P_2_W_19_Ag^I^O_70_H_2_]^13–^, which has a total valence of Meyer bond order^37^ as 0.94, significantly higher than the Meyer bond order sum of five strongest Ag^+^-O bonds shown in Figure 1d (0.65).^38^

**Figure 2:**
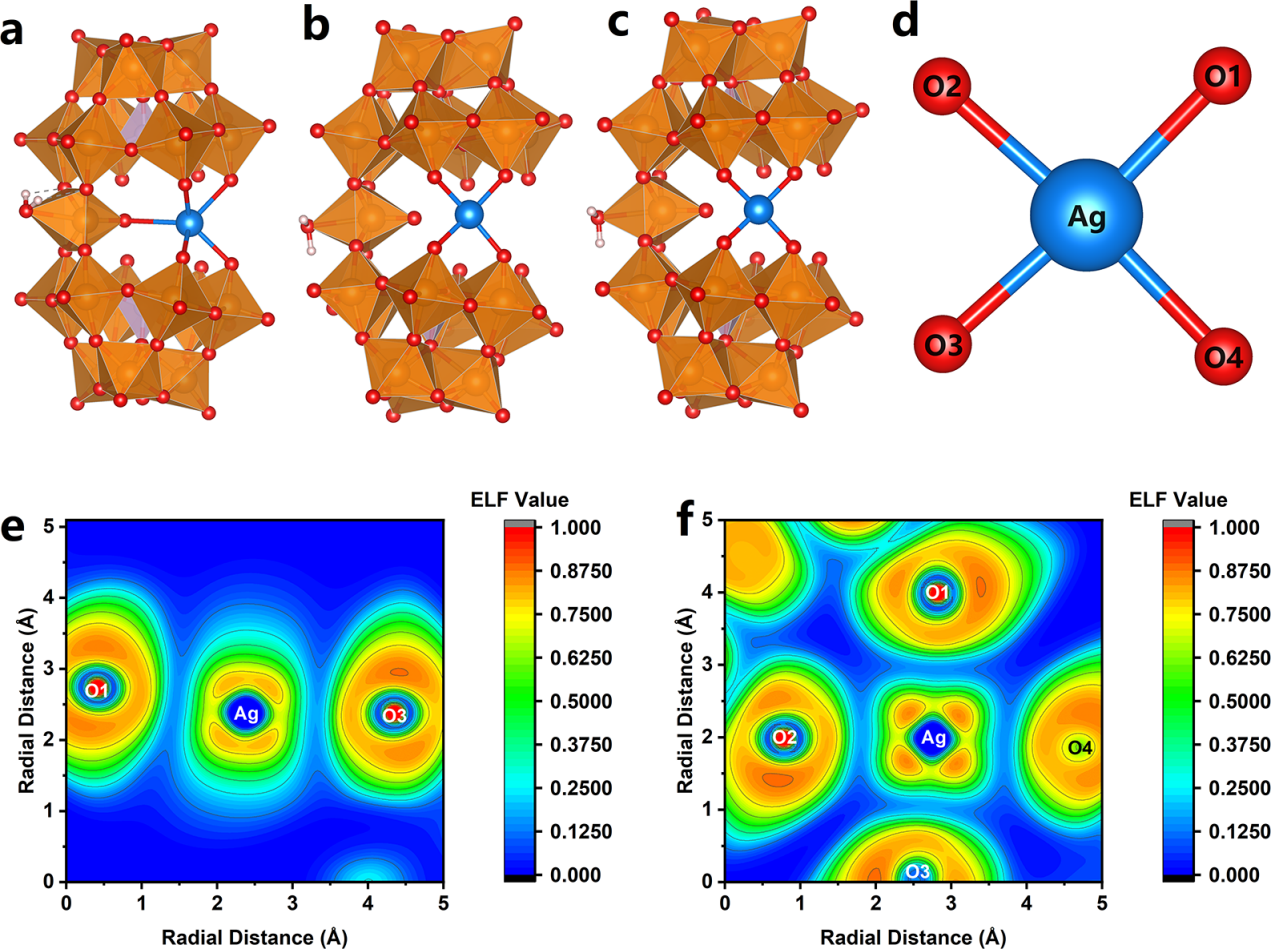
Computationally optimized geometric structure of [P_2_W_19_Ag^I^O_70_H_2_]^13–^ (a), [P_2_W_19_Ag^II^O_70_H_2_]^12–^ (b), and [P_2_W_19_Ag^III^O_70_H_2_]^11–^ (c). Calculated coordination geometry of Ag^3+^ center in [P_2_W_19_Ag^III^O_70_H_2_]^11–^ with atoms used for ELF analysis labeled (d). ELF mapping of the Ag^III^–O bonds along the O1–Ag–O3 plane (e) and the O1–Ag–O2 plane (f).

The oxidation state of Ag in **1** is also studied by X-ray photoelectron spectroscopy measurement, from which the oxidation state of Ag can be reflected by the binding energy (BE) of the photoelectrons. Upon reduction of the sample, it is found that the Ag 3d peaks shift to a lower BE, which suggests a decrease in the content of an oxidative component with a higher chemical shift. By fitting the experimental spectra with two sets of peaks, a higher 3d_5/2_ component is found at 368.4 eV, lying approximately 0.6 eV higher than another component (367.8 eV) and can be attributed to silver at higher oxidation states. The lower set of peaks may thus be attributed to Ag^+^. This trend is also in line with some other reported Ag^3+^ complexes.^39–41^ Upon reduction, the peak intensity of the Ag^+^ component increased significantly (Figure S8). It is noteworthy that these peaks do not show significant broadening as observed in the case of Ag^2+^,^42^ further confirming their assignment as Ag^+^ and Ag^3+^, both of which tend to have narrower peaks. A similar phenomenon is observed in the electron energy loss spectroscopy (EELS) measurements on **1**, where the element with a higher oxidation state usually shows higher energies of energy loss edges.^43^ EELS results exhibit an 1–2 eV redshift of the Ag M_4,5_ energy loss edge after reduction, with the edge onset energy lowering from 413.5 eV in the fresh samples to approximately 412.5 eV in the case of partially reduced samples, suggesting a lowering in the oxidation state (Figure S9). This can be explained by the loosening of the binding of inner (3d) electrons of Ag when being reduced from Ag^3+^ to Ag^+^. These results together further suggest the existence of the high-valent Ag^3+^ complex in **1.**

The UV–vis spectrum of **1** in aqueous solution as seen in Figure 3a shows a characteristic intense absorption peak at 200 and 260 nm assigned to the O^2–^-to-W^6+^ charge-transfer characteristic for polytungstates containing lacunary Keggin-type moieties.^5^ Another absorption peak is at 420 nm, which decreases significantly after 24 h with the fading of the solution as depicted in Figure 3b, which is almost identical to the aqueous solution UV–vis spectrum of reduced samples of **1** (Figure S10) containing mainly H_*x*_[P_2_W_19_Ag^I^O_70_H_2_]^(13–*x*)–^. Detailed time-dependent UV–vis measurements confirmed the gradual decrease in the intensity of the 420 nm peak within a 45 min measurement period (Figure 3c). This suggests that this peak may be the O^2–^-to-Ag^3+^ ligand-to-metal charge transfer (LMCT) absorption of the high-valent Ag^3+^,^44^ and its decrease corresponds to the spontaneous reduction of Ag^3+^ to Ag^+^ in aqueous environments. The peaks in the UV region meanwhile remained almost unchanged, indicating that the structure of the POM framework was not significantly affected during this process (Figure 3a), indicating its structural robustness. Time-dependent density functional theory (TD-DFT) calculations revealed the lowest strong transitions of [P_2_W_19_Ag^III^O_70_H_2_]^11–^ at around 400–460 nm (see Figure 3d), close to the experimentally observed absorption peak in the visible region. This LMCT feature is further confirmed by the simulated distribution of photoelectrons and holes generated by the corresponding excitations^45^ (Figure 3f–i). Meanwhile, the lowest transitions of [P_2_W_19_Ag^I^O_70_H_2_]^13–^ (Figure 3e) all fell into the wavelength range shorter than 300 nm without any observable transitions in the visible light region, which confirms that the 420 nm peak observed in the UV–vis spectra can only be assigned to the LMCT charge transfer of Ag^3+^ in compound **1.**

**Figure 3:**
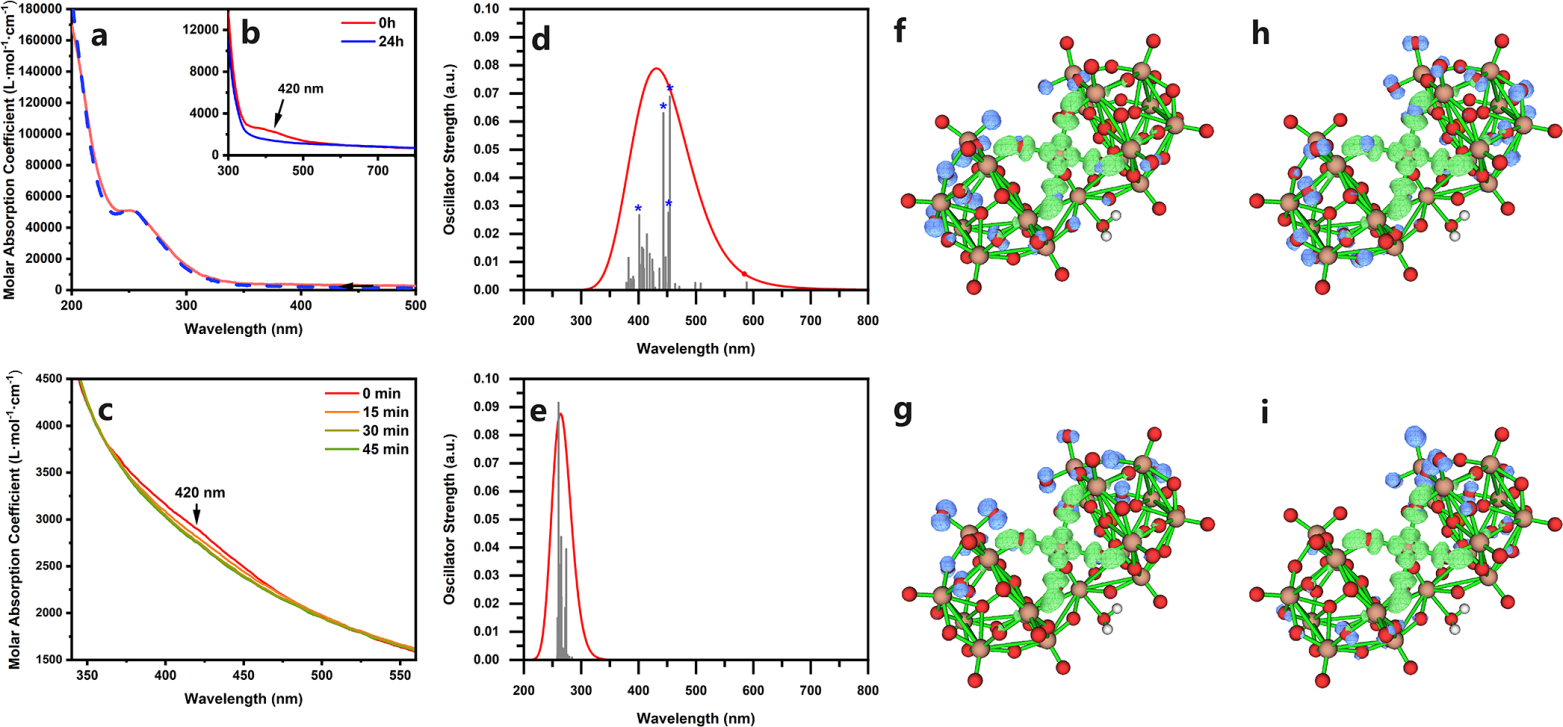
Solution UV–vis spectrum of **1** in the wavelength range of 200–500 nm (a) and 300–800 nm (b) showing the difference between freshly prepared solution (red lines) and the same solutions after 24 h (blue lines). Detailed time-dependent UV–vis spectrum showing the decrease in absorption of the peak at 420 nm (c). Lowest transitions (gray lines) and corresponding simulated absorption curve (red lines) of [P_2_W_19_Ag^III^O_70_H_2_]^11–^ (d) and [P_2_W_19_Ag^I^O_70_H_2_]^13–^ (e) obtained by TD-DFT calculations. The electron–hole distribution corresponding to four transitions of [P_2_W_19_Ag^III^O_70_H_2_]^11–^ with the highest oscillator strengths (8th, 9th, 12th, and 23rd marked with asterisks in Figure 3d) are shown in Figure 3f–i in the sequence of transition energy. Isosurfaces of photoelectrons and holes are shown in green and blue, respectively; an isosurface value of 0.002 was chosen for all isosurfaces.

The cyclic voltammetry of purified **1** in 0.5 M NaNO_3_ solution was conducted. Figure 4a shows a pair of redox peaks at approximately 1.67 and 1.70 V vs NHE, respectively. The peak current of the two peaks suggests a quasi-reversible process, and the difference between peak potentials is observed to be ca. 30 mV, very close to the expected value for a two-electron quasi-reversible redox process (56.5 mV/n for n-electron reversible redox reactions, where *n* = 2 in our case)^46^ that can be attributed to the Ag^3+^/Ag^+^ two-electron redox pair. The reduction peak current seems always smaller, suggesting a quick in situ reduction of the electrochemically formed Ag^3+^ species, which lowers the Ag^3+^ concentration and weakens the reduction current,^47^ in line with the expected strong oxidation ability of [P_2_W_19_Ag^III^O_70_H_2_]^11–^. The peak current is proportional to the square root of the scan rate (Figure 4b,**c**), which confirms that the reaction rate is diffusion-controlled and the difference in peak potentials reflects the number of electrons transferred.^47^ Further rinse tests showed no indications of peaks attributed to **1** after rinsing and measuring the CV diagram in blank solutions (Figure 4d), excluding the possible adsorption or electrodeposition of any active Ag-containing species on the electrode. At higher potentials, the catalytic water oxidation current can be observed. Interestingly, no electrochemical redox process related to W^6+^ was observed in the cyclic voltammogram (CV) of **1** (Figure S11). This is consistent with the expected properties for POM anions with *cis-*WO_2_ moieties, which generally do not show reversible W^6+^/W^5+^ peaks.^48^ Meanwhile, it has been reported that the W^6+^/W^5+^ redox peaks of some phosphotungstates may be difficult to observe under higher pH.^49^ Thus, the low solubility of **1** and the rather high pH (∼7) of the solution may have also contributed to the absence of W^6+^-related peaks. The characteristic oxidation peak of **1** remains unchanged after an electrolysis test under 1.85 V vs NHE for 1800 s (See Figure S12), indicating the stability of the POM-encapsulated Ag^3+^/Ag^+^ redox pair in **1** under applied conditions. This stability may be explained by the chelating effect of POMs on Ag^3+^ and Ag^+^ through their highly negatively charged cavity.

**Figure 4:**
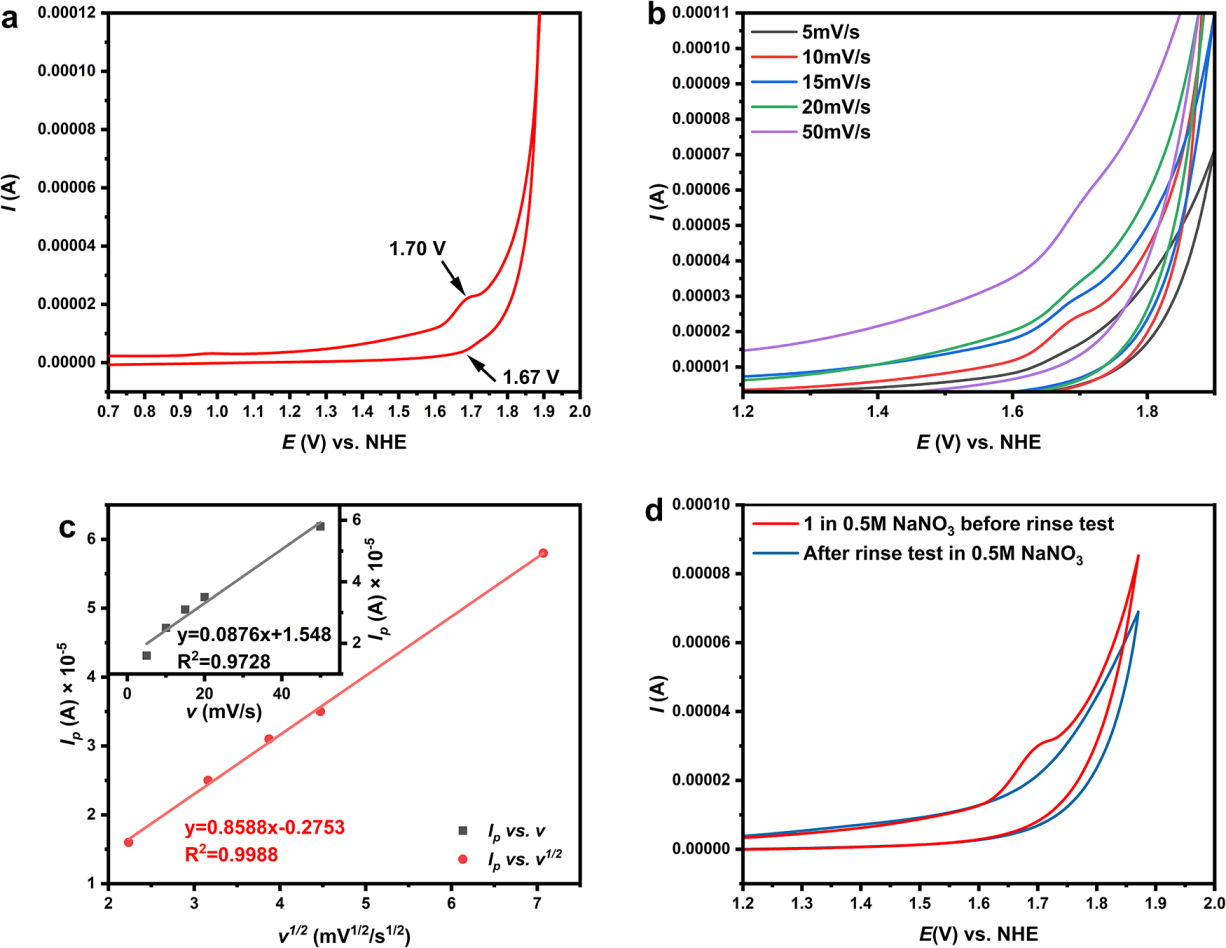
(a) CV of **1** in the range of 0.7–2.0 V vs NHE. (b) Scan rate-dependent CVs of compound **1**. (c) Different correlations between the oxidation peak current *I*_p_ and the scan rate *v*. (d) Rinse test CV of the working electrode. Working electrode: glassy carbon; reference: Ag/AgCl (saturated); scan rate: 5 mV/s for Figure 4a,d.

In comparison, the CV of **1** resembles the similar redox process of AgNO_3_ under the neutral aqueous environment as displayed in Figure S13 which shows a pair of well-separated redox peaks at 1.41 and 1.57 V vs Ag/AgCl. This process forms solid Ag^I^Ag^III^O_2_ as the main product,^50^ the deposition of which changes the system from homogeneous to heterogeneous states, different from the case of **1** which remains in a homogeneous state; meanwhile, the very large difference between the peak potentials in this case as well as the significantly asymmetrical shape of the two peaks indicate a rather low electrochemical reversibility for the process as compared with **1**. The CV behaviors of the high-valent Ag species have been shown to be highly structure-dependent.^9,50,51^ Given the instability of free Ag^3+^ in aqueous media,^49^ this difference in the CV confirms the successful stabilization of Ag^3+^ homogeneously within the POM framework, leading to a different electrochemical behavior compared to free Ag^+^ ions in AgNO_3_ solution that forms heterogeneous oxides through electrochemical oxidation. The characteristic CV curve of **1** remaining unchanged in the different tests illustrated above is also in line with its structural integrity and durability in the electrochemical processes.

It is well-known that the electrochemical reversibility reflects the rate of the electron-transfer process, based on which we may expect a rather quick electron transfer for the Ag^3+^/Ag^+^ redox pair stabilized in **1**, highlighting their potential as catalytic active “electron shuttles.” It is further confirmed by electrochemical impedance spectrometry results with a much smaller charge-transfer resistance (*R*_CT_) under the presence of **1** in the solution (Figure S14) compared with the blank electrolyte solution. This can be explained by the Marcus theory, based on which the barrier for the electron-transfer processes is relevant to both structural change and the energy difference between the reactant and the product.^52^ The kinetic barrier of these POMs can be evaluated based on the activation energy of self-exchange electron-transfer reactions, while the relatively small change in coordination geometries of the Ag centers between different oxidation states resulted in a relatively small overall Marcus internal reorganization energy value^52,53^ Taking an estimated value of ∼18 kcal/mol for the external reorganization energy based on previous results on POMs,^54,55^ the barriers of the two steps of reduction are all considerably small (<20 kcal/mol; see Figure S15, Tables S6 and S7), indicating that the electron-transfer process can happen very quickly almost without any significant activation required. It is noteworthy that **1** provided a rare example of electron shuttles with a rather high redox potential, highlighting its potential use as a redox catalyst under strongly oxidative conditions. The structural integrity of the POM, benign hostage, and accessibility by small substrate(s) of this opened framework to stabilize various silver ions may offer stable redox catalysis within the framework.

Given the fact that **1** is isolated from the same mother liquor where the Ni^3+^-containing POMs were obtained, it can be postulated that fast electron transfer of the Ag^3+^/Ag^+^ redox pair in **1** seemed to have played a crucial role in accelerating the oxidation of POM-embedded metal centers by peroxydisulfates. To further probe their catalytic properties in redox reactions, such as the catalytic efficiency and versatility on different substrates, we tested the catalytic activity of **1** under electrochemical conditions using catalytic oxidative benzylic C–H activation as the model reaction. This reaction is considered a promising route to yield important chemicals by oxidizing substrates in an environment-friendly manner (e.g., oxidizing toluene to yield benzyl alcohol and benzaldehyde), and the source of oxygen would be either derived from water or oxygen in the air without any addition of external waste-generated oxidants.^56^

Electrolysis of 4 mM toluene in 10 mL of 0.5 M NaNO_3_ solution under 1.65 V vs Ag/AgCl in a period of 1800 s could result in benzaldehyde and benzyl alcohol as the main products. The Faradaic efficiency (FE) of this reaction is however very low, and bubbles are observed on the surface of electrodes during the electrolysis process, suggesting the possible competition between C–H oxidation reactions and the electrolysis process of water. Given the fact that the potential applied here is in the electrochemical water oxidation region (see Figure 4a), it can be estimated that water oxidation would be the main side reaction that affects the overall FE. Upon the addition of **1** as the electrocatalyst to the reaction mixture, the electrolytic current density increased by approximately 45%, confirming the acceleration of electron transfer by **1** as the electron shuttle, as monitored by chronoamperometry (Figure S16). The stable current also confirms the stability of **1** in the reaction during the electrolysis period, with an initial decrease of current corresponding to the local concentration polarization on the electrode surface, which is commonly observed in almost all CA curves.^57^ Interestingly, a significant increase in both overall conversion and FE was observed (see Table 1), revealing a current specificity for the toluene oxidation to products that is higher than that for the water oxidation reaction. We attribute this effect to the role played by the negatively charged POM ligands in [P_2_W_19_Ag^III^O_70_H_2_]^11–^ through electrostatic stabilization of the charged carbocation intermediates formed in the reaction pathway.^56,58^ This clearly suggests the potential activity of **1** toward the electrochemical oxidation of toluene in the aqueous phase, similar to previously reported results for other substituted POMs.^17,58^ The products were analyzed by GC-MS (Figures S17–S19), and no benzoic acid or corresponding Kolbe decarboxylation products were observed as the byproducts of the reaction.

**Table 1:**
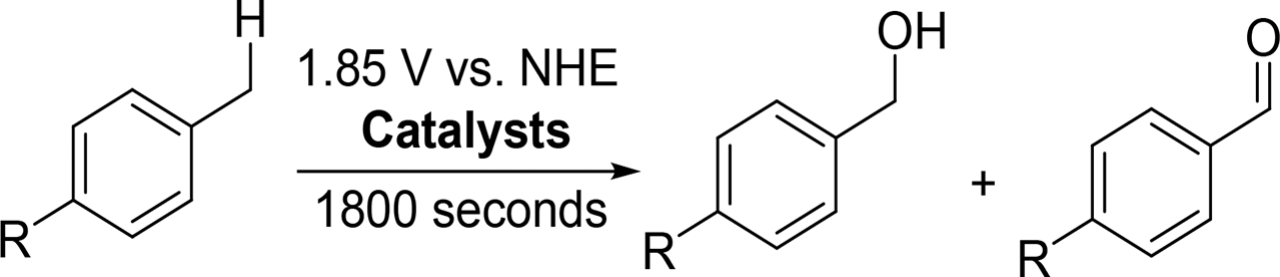
Results of Electrochemical Oxidative Benzyl C–H Activation of the Substrates Tested

substrate	conversion (mol %)	FE (%)	alcohol selectivity (mol %)	aldehyde selectivity (mol %)
toluene^a^	3	18	73	27
toluene^b^	21	58	75	25
toluene^c^	3	23	80	20
4-chlorotoluene^a^	2	8	49	51
4-chlorotoluene^b^	7	24	49	51
p-xylene^a^	5	42	58	42
p-xylene^b^	29	85	64	36

a Reactions conducted in 0.5 M NaNO_3_ solution. Working electrode: Pt plate (*S*_eff_ = 4 cm^2^). Reference electrode: Ag/AgCl (saturated KCl). Counter electrode: Pt wire. No catalyst added to the reaction mixture.

b 1.0 mg (0.16 μmol) of **1** was added as the catalyst.

c 0.8 mg (0.32 μmol) of Na_9_[PW_9_O_34_]·13H_2_O was added as the catalyst.

Measurements on hydrogen kinetic isotope effects revealed a KIE value of approximately 2.7–2.8, indicating that the reaction mainly proceeds through a proton-coupled electron transfer (PCET) mechanism.^17^ Notably, similar PCET mechanisms are also reported for catalytic electrochemical oxidation reactions of other toluene derivatives by POMs.^56^ This indicates that the reaction proceeds through steps of one-electron PCET reduction of Ag^3+^ in [P_2_W_19_Ag^III^O_70_H_2_]^11–^ to Ag^+^, where the Ag^2+^ species are expected to form as intermediates. Correspondingly, the absence of Ag^2+^ from the experimental evidence as discussed above may be presumably due to its instability compared with the Ag^3+^ and Ag^+^ species. Interestingly, approximately the same KIE value was also obtained for reactions without the presence of **1**, indicating that the addition of **1** does not alter the reaction mechanism, further confirming that the catalysis of **1** is based on accelerating the electron-transfer process rather than changing the reaction pathway. The proposed mechanism of oxidation is shown in Scheme 1a. This is further confirmed by expanding the reaction to different *para*-substituted toluene derivatives as shown in Table 1, revealing a pattern of increase in FE along with the increase of electron-donating ability of the *para* substituents.

**Scheme 1:**
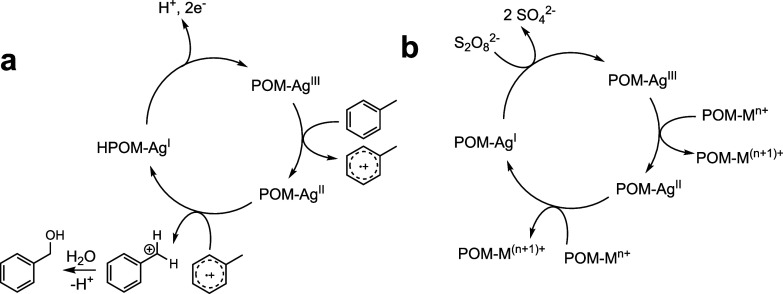
Proposed Mechanism of (a) Oxidation of Toluene to Form Benzyl Alcohol Catalyzed by **1** and (b) Oxidation of POMs by Silver-Assisted Persulfate Oxidation Method

In light of the above study, the facile redox transformation between [P_2_W_19_Ag^III^O_70_H_2_]^11–^ and [P_2_W_19_Ag^I^O_70_H_2_]^13–^ can also further support an envisaged catalytic cycle in our reported Ag^+^-promoted peroxydisulfate oxidation method for the preparation of other high-valent transition-metal species in the POM framework.^16,22^ Thus, there is a purely inorganic catalytic cycle without fragile organic moieties whereby the high-valent Ag-containing POM functions as a “catalyst bearing catalysts” toward the construction of other transition-metal high-valent POMs. The proposed catalytic mechanism is depicted in Scheme 1b.

## Conclusions

In this work, a high-valent Ag^3+^-containing POM **1** is identified with subsequent characterizations, showing its unique electronic structure and potential catalytic activity toward electrochemical C–H activation reactions based on its high intrinsic redox potentials and the ability of accelerating electron-transfer reactions. In particular, we show that the POM functions not only as a robust, recyclable oxidation-resistant ligand, protecting the Ag species from disintegrating, but also as a source of stabilization toward positively charged intermediates during the redox catalysis. The reaction-specific catalytic activity of **1** seems similar to some biological redox-active compounds and enzymes, which may be further regarded as a “biomimetic” redox catalyst.^59^ The results obtained from this study may help us to develop more active oxidative catalysts based on Ag-POM systems. It can be postulated that more compounds containing high-valent Ag^2+^ and Ag^3+^ may be synthesized with the stabilization of these oxidative centers by the robust, oxidation-resistant POM ligands and that the redox properties of the high-valent Ag centers may be “rationally” modified by changing the structure of the POM framework.

## Supplementary Material



## Notes

Experimental section, synthetic procedure, general characterization methods, computational studies, single-crystal XRD measurement, electrochemical measurement, and catalytic test, and additional references 60–66 (PDF)
