# Supplementary material for: Switchable High-Valent Ag3+/Ag+ Redox Pair Stabilized in Polyoxometalate as Highly Oxidative “Electron Shuttle” Catalysts
Source: JACS Au. 2025 Oct 14;5(10):5089–97. doi: 10.1021/jacsau.5c00987 (PMC12569697; doi:10.1021/jacsau.5c00987)
Supplement: Supplementary file 1 [file au5c00987_si_001.pdf]

Electronic Supporting Information

Switchable High-valent Ag^{3+}/Ag^{+} Redox Pair Stabilized in a Polyoxometalate as Highly Oxidative "Electron Shuttle" Catalysts

Xiang Li,[a] Hehua Hui,[b] Yuanhang Ren,[b] Zhaoqing Liu,[c] Yiyang Li*,[a] Bin Yue*,[b] Heyong He*[b] and Shik Chi Edman Tsang†[a]

[a] Department of Chemistry, University of Oxford, Oxford, OX1 3QR, United Kingdom

[b] Department of Chemistry and Shanghai Key Laboratory of Molecular Catalysis and Innovative Materials, Fudan University, Shanghai 200438, China

[c] College of Chemistry and Molecular Engineering, Peking University, Beijing 100871, China

*Corresponding authors: yiyang.li@chem.ox.ac.uk
yuebin@fudan.edu.cn
heyonghe@fudan.edu.cn

Table of Contents

**Experimental Section**	**5**
Synthetic Procedure	**5**
General Characterization Methods	**5**
Computational Studies	**6**
Single Crystal X-ray Diffraction Measurement	**7**
Electrochemical measurement	**7**
Catalytic activity test	**8**
**Figure S1.** Typical crystallites of **1** with different size (left) and the illustration on the separation of **1** from $Cs_4K[PW_9Ni^{III}{}_2Ni^{II}O_{40}H_8]\cdot 6H_2O$ (right) in sample vials used for crystallization.	**9**
**Figure S2**. Thermogravimetric curve of **1**.	**9**
**Figure S3**. Schematic illustration of the crystal structure of **1** showing thermal ellipsoids.	**9**
**Figure S4**. Comparison of (a) IR spectra and (b) Raman spectra of freshly-prepared and partially reduced samples of **1**.	**10**
**Figure S5**. EPR spectrum of solution and solid samples of **1** under 300K.	**10**
**Figure S6.** Molecular orbital (MO) analysis of $[P_2W_{19}Ag^{III}O_{70}H_2]^{11-}$. (**a**) The MO energy level diagram of $[P_2W_{19}Ag^{III}O_{70}H_2]^{11-}$, with MOs represented as light grey bars. The contributions from Ag 4d orbital components to each molecular orbital are illustrated as the relative length of the red bars. (**b**) Visualized distribution of frontier molecular orbitals of $[P_2W_{19}Ag^{III}O_{70}H_2]^{11-}$.	**11**
**Figure S6.** Molecular orbital (MO) analysis of $[P_2W_{19}Ag^{I}O_{70}H_2]^{13-}$. (**a**) The MO energy level diagram of $[P_2W_{19}Ag^{I}O_{70}H_2]^{13-}$, with MOs represented as light grey bars. The contributions from Ag 4d orbital components to each molecular orbital are illustrated as the relative length of the red bars. (**b**) Visualized distribution of frontier molecular orbitals of $[P_2W_{19}Ag^{I}O_{70}H_2]^{13-}$.	**12**
**Figure S8**. X-ray photoelectron spectra of (**a**) fresh sample and (**b**) partially reduced sample of **1**. Peak positions are labelled in the figures.	**13**
**Figure S9**. Electron energy loss spectra of fresh sample (blue) and reduced sample (red) of **1**. Corresponding 15 points adjacent-averaging smoothed data are shown to highlight the edge profile.	**13**
**Figure S10**. UV-Vis spectra of reduced sample of **1** in aqueous solution (red line) and solution of fresh sample of **1** after 24 hours (blue line).	**14**

**Figure S11**. Cyclic voltammogram of (**a**) overall scan of **1** in the range of -1.0~1.9 V vs. NHE and (**b**) detailed scan in the range of -1.0 ~ 0.2 V vs. NHE under a scan rate of 5 mV/s.	**14**
**Figure S12**. (a) Chronoamperometric curve of **1** in 0.5 M $NaNO_3$ solution under 1.85 V vs. NHE for 1800 s using a glassy carbon electrode (S=0.07cm^2) as the working electrode. (b) Cyclic voltammogram of **1** before and after the corresponding electrolysis test. The increase in current after electrolysis test under 1.85 V vs. NHE may be attributed to the change in surface status of the glassy carbon electrode due to oxidation.	**15**
**Figure S13**. Cyclic voltammogram of $AgNO_3$ in the range of 0.7~2.0 V vs. NHE. Working electrode: glassy carbon; reference: Ag/AgCl (saturated); Scan rate: 5 mV/s.	**15**
**Figure S14**. Electrochemical impedance spectra of the glassy carbon electrode in 0.5 M $NaNO_3$ with (blue) and without (red) **1**. Fitting results corresponding to the equivalent circuit shown in the figure are shown as lines.	**16**
**Figure S15**. Schematic illustration of the calculation of Marcus internal reorganization energy (λ_i) based on Nelson's method, taking $[P_2W_{19}Ag^{III}O_{70}H_2]^{11-}$/$[P_2W_{19}Ag^{II}O_{70}H_2]^{12-}$ redox pair as an example. In this figure, E1 and E3 represent the energy of $[P_2W_{19}Ag^{II}O_{70}H_2]^{12-}$ and $[P_2W_{19}Ag^{III}O_{70}H_2]^{11-}$ under their optimised structures, E2 the energy of $[P_2W_{19}Ag^{II}O_{70}H_2]^{11-}$ with the same geometry as the optimised structure of $[P_2W_{19}Ag^{III}O_{70}H_2]^{12-}$, and E4 the energy of $[P_2W_{19}Ag^{III}O_{70}H_2]^{12-}$ with the same geometry as the optimised structure of $[P_2W_{19}Ag^{II}O_{70}H_2]^{11-}$. The overall λ_i value of the self-exchange reaction can be calculated based on the formula λ_i=|E2-E1|+|E4-E3|.	**17**
**Figure S16.** Typical chronoamperometric curve of the electrochemical oxidation of toluene with (blue) and without (red) the presence of **1**, corresponding to the condition b and a in **Table 1**, respectively.	**18**
**Figure S17.** GC-MS mass spectra of chlorobenzene (internal standard) and the substances occurring in the electrochemical oxidation of toluene, with the *m/z* value of peaks labelled in blue. Background peaks from the small amount of air and water are marked with asterisks. Note that in the mass spectra of some products, these are relatively more significant due to the low concentration of the substrate.	**18**
**Figure S18.** GC-MS mass spectra of the substances occurring in the electrochemical oxidation of xylene, with the *m/z* value of peaks labelled in blue. Background peaks from the small amount of air and water are marked with asterisks.	**19**
**Figure S19.** GC-MS mass spectra of the substances occurring in the electrochemical oxidation of 4-chlorotoluene, with the *m/z* value of peaks labelled in blue. Background peaks from the small amount of air and water are marked with asterisks.	**19**
**Table S1.** Crystal data of **1**.	**20**
**Table S2.** BVS calculation of selected atoms in **1**.	**21**

**Table S3.** Cartesian structural coordinates of optimized structure of $[P_2W_{19}Ag^{III}O_{70}H_2]^{11-}$.	**21**
**Table S4.** Cartesian structural coordinates of optimized structure of $[P_2W_{19}Ag^{II}O_{70}H_2]^{12-}$.	**25**
**Table S5.** Cartesian structural coordinates of optimized structure of $[P_2W_{19}Ag^{I}O_{70}H_2]^{13-}$.	**29**
**Table S6.** Calculated relative energies used for the Marcus internal reorganization energy (λ_i) calculation based on Nelson's four-points method, comparing to the E1 of each redox pair (all relative E1 value are set to 0 kcal/mol).	**33**
**Table S7.** Calculated Marcus internal reorganization energy (λ_i) of the $[P_2W_{19}Ag^{III}O_{70}H_2]^{11-}$/$[P_2W_{19}Ag^{II}O_{70}H_2]^{12-}$ and $[P_2W_{19}Ag^{II}O_{70}H_2]^{12-}$/$[P_2W_{19}Ag^{I}O_{70}H_2]^{13-}$ redox pairs based on Nelson's four point method, the standard electrode potential (E^{Θ}) values of Ag(III)/Ag(II) and Ag(II)/Ag(I) redox pairs, and the corresponding Gibbs free energy value (ΔG^{Θ}).	**33**
**References**	**34**

Experimental Section

Synthetic Procedure

The polyoxometalate precursor $Na_8HPW_9O_{34}\cdot 13H_2O$ was synthesized based on synthetic procedure reported in previous literatures.[1] All other reagents used in the synthesis were purchased from Sigma Aldrich. For a typical synthetic procedure, 2.2 g (0.82 mmol) $Na_9PW_9O_{34}\cdot 13H_2O$ and 300 mg (1.0 mmol) $Ni(NO_3)_2\cdot 6H_2O$ were mixed in 15 mL of deionized water. The mixture was heated to 70°C to dissolve all solids, followed by addition of 500 mg (0.19 mmol) $Na_9PW_9O_{34}\cdot 13H_2O$, 42 mg (0.25 mmol) CH_3CO_2Ag and 1.65 g (6.1 mmol) $K_2S_2O_8$ with continuous stirring under 70°C till the solution turned brownish black. The solution is then further stirred under room temperature for 10 mins, and 2.0 g (10.3 mmol) $CsNO_3$ was then added. The solution was further stirred under room temperature for 20 minutes, resulting in a suspension containing a large amount of brownish precipitate. The solid raw product was separated from the mixture by centrifugation and redissolved in 25 mL of deionized water under 50°C. The solution was then kept under this temperature without stirring for sedimentation. The resulted dark brown supernatant was then transferred into glass vials and left under room temperature for crystallization purpose. After 3~4 days, the resulted dark orange crystals of $Cs_7K_4[P_2W_{19}Ag^{III}O_{69}(OH_2)]\cdot 17H_2O$ (**1**) were collected and dried in air (8 mg, 0.5% based on Ag). Due to the low yield of the product (with part of the tungstate forming $Cs_4K[PW_9Ni^{III}{}_2Ni^{II}O_{40}H_8]\cdot 6H_2O$), sufficient amounts of **1** used for characterizations were obtained *via* multiple preparation experiments conducted at the same time. IR: 1083(s), 1071(m, sh), 1019(s), 948(s), 917(m), 893(w, sh), 849(m, sh), 754(m). Elemental Analysis (wt%) result: Calcd: W, 57.21; Cs, 15.24; Ag, 1.77; K, 2.56; P, 0.51. Found: W, 57.15; Cs, 15.44; Ag, 1.96; K, 2.56; P, 0.58.

General Characterization Methods

The IR spectra were recorded by attenuated total reflectance measurements a Nicolet iS50 FT-IR spectrometer with Smart Golden Gate Accessory with ZnSe lenses. The UV-Vis spectra were recorded by a Shimadzu UV-2600 UV-Vis spectrometer in the range of 200~800 nm. The concentration of the solution used for the measurement is 66 μmol/L (for time-dependent UV-Vis measurement in the range of 350~450nm), 24 μmol/L (for the measurement in the range of 300~800nm) and 5 μmol/L (for the measurement in the range of 200~500nm), respectively. Thermogravimetric experiments were performed on a TA instruments Q600 SDT Thermogravimetric analyzer under air atmosphere (flow rate: 100.0 ml/min, heating rate: 10.00 °C/min) in the temperature range of 30~900°C with the starting mass of 5.229 mg. Continuous-wave EPR measurement were conducted on an X-band (9.4 GHz) Bruker EMX EPR

spectrometer over a 1000 Gauss field range under 293K and 77 K. ICP-MS elemental analysis of the compound (for P, W, K and Ag) was performed on an Perkin Elmer NexION 2000B ICP-MS instrument External calibration analysis method was used to obtain the concentration of relevant elements. EDX elemental analysis (for Cs, P and Ag) was performed on a Phenom Prox Scanned Electron Microscope. X-ray photoelectron spectrum (XPS) was recorded by a PerkinElmer PHI 5000C ESCA system in the range of 0-1200 eV. Al K_{α} (hν = 1486.6 eV) was applied as the irradiating X-ray. The aluminium anode tube for the X-ray emission was operated at a voltage of 12 kV and kept constant during all measurements. Scans were obtained at a pass energy of 40 eV, 10 scans with step size 0.1 eV. All the binding energy values are calibrated by using C 1s = 284.6 eV as a reference. Electron energy loss spectrum (EELS) was recorded on a Talos F200X G2 Field-emission transmission electron microscope with a Gatan Continuum S/1077 EELS spectrometer, under the collection semi-angle of 100 mrad, the convergence semi-angle 0 mrad and the dispersion (eV/channel) of 0.3. The drift tube voltage was 320.0 V. The Energy Loss Near-Edge Structure (ELNES) analysis, including the zero-point calibration, baseline subtraction and data normalization, was performed using Gatan DigitalMicrograph 3.6.2 software pack. The onset energy of the energy loss edge was defined as the energy loss value where the intensity reaches 10% of the adjacent peak (at approximately 425 eV).

Computational Studies

All computational works were performed on by Density Functional Theory (DFT) methods using Gaussian 16 computational software pack[2] on University of Oxford Advanced Research Computing (ARC) facilities.[3] PBE0 hybrid functional with DFT-D3BJ (Becke-Johnson) dispersion correction were used for all calculations, and IEFPCM solvation model was applied to all calculations with water as the solvent.[4,5] Geometric optimization were performed using 6-31G(d)/lanl2dz basis sets. Specifically, 6-31G(d) basis set was used for O, P and H atoms while lanl2dz basis set was applied to all metal atoms. All structures are optimized to local minimum without imaginary frequencies.[6-10]

The Time-Dependent Density Functional Theory (TD-DFT) analysis on the UV-Vis features were performed under PBE0-D3BJ/def2tzvp computational level.[11] 30 lowest transitions were calculated for all selected anions. Electron-hole analysis[12] of the UV-Vis spectrum and LOBA analysis[13] on the oxidation state of Ag centres was conducted using Multiwfn 3.8 software pack.[14]

The Marcus activation energy[15] is calculated based on the following equation:

$$E_a = \frac{(\lambda + \Delta G°)^2}{4\lambda}$$

Where E_a is the activation Gibbs free energy, $\Delta G°$ the thermodynamic Gibbs free energy difference between the reactants and the products (where $\Delta G°=0$ for a self-exchange reactions), and λ the reorganization energy that represents the contribution of structural changes, both the reactant molecules and the solvent spheres, to the activation barrier.[22][] The reorganization energy can be further decomposed into two terms as shown below:

$$\lambda = \lambda_e + \lambda_i$$

Where λ_i the "intrinsic" reorganization energy corresponding to the structural change of reactant molecules. This term can be calculated by using Nelson's method[16] (See **Figure S15** and **Table S7**). λ_e is the solvent reorganization energy, corresponding to the change in the solvation structures of the reactants, for which a value of ~18 kcal/mol has been observed for polyoxometalate species experimentally.[17,18]

Single Crystal X-ray Diffraction Measurement

Collection of crystal diffraction data for compound **1** was performed on a Bruker SMART APEXII single crystal diffractometer with graphite-monochromated Ga-K_α radiation (λ = 1.34139 Å). Data of linear absorption coefficients, atomic scattering factors and anomalous dispersion corrections were obtained from International Tables for X-ray Crystallography. Empirical absorption was applied to the absorption correction. The crystal structure of **1** was resolved by direct method and refined by full-matrix least-squares method on F^2 on SHELXL-2018/3 crystallographic software package.[19] All atoms were refined anisotropically. The visualization of crystal structure was performed using VESTA 3.5.8 software pack.[20]

It should be noted that other possible identities of Ag2 atom as Na^+ or K^+ (based on the bond length) can be safely excluded by the unreasonably large occupancies when refined as the corresponding atoms (>1) and the close distance between Ag1 and Ag2 sites, which further disallow the total occupancy of Ag1 and Ag2 atoms being larger than 1.

Electrochemical measurement

Electrochemical measurements were performed in 0.5 M $NaNO_3$ solution in a three-electrode electrochemical cell on an Ivium VERTEX electrochemical workstation. an Ag/AgCl (saturated KCl) electrode was used as the reference electrode and Pt wire electrode was used as the counter electrode. Unless otherwise stated, a glassy carbon with an effective surface area of 0.07 cm^2 was used as the working electrode for general cyclic voltametric tests. Electrochemical

impedance spectroscopy (EIS) measurements were conducted in the frequency range of 0.1 Hz to 100 kHz with an AC voltage of 10 mV amplitude. Data fitting was conducted using EIS Spectrum Analyser software pack.[21]

Catalytic activity test

All electrochemical catalytic tests were performed in a three-electrode electrochemical cell on an Ivium VERTEX electrochemical workstation. an Ag/AgCl (saturated KCl) electrode was used as the reference electrode and Pt wire electrode was used as the counter electrode. A platinum plate electrode (4 cm^2 effective area) was used as the working electrode. Typically, a mixture of a given amount of catalysts, 40 μmol chlorobenzene (internal standard for GC-MS analysis) and 40 μmol substrate in 10 mL of 0.5 M $NaNO_3$ solution were stirred continuously and electrolyzed under constant potential (1.65 V vs. Ag/AgCl) for 1800 seconds. The mole of electrons passed through the electrochemical cell was determined by chronoamperometric measurements. After reaction, the pH of the solution was adjusted to 2 by 1M nitric acid to acidify possibly formed carboxylic acid products, followed by addition of 2 mL diethyl ether to extract all organic components in the reaction mixture. The resulted organic extracts were dried with anhydrous Na_2SO_4 and analyzed in an Agilent 5977A Gas Chromatograph-Mass Spectrometer. The conversion and selectivity of the catalytic reactions were determined by internal standard method. The chromatographic peaks were assigned based on their mass spectra using the MSD ChemStation Enhanced Data Analysis software. The Faradic efficiency of the reaction was obtained by diving the mole of electrons required to form products (2 for alcohols and 4 for aldehydes) by the total amount of electrons passing through the circuit.

An 1:1 mixture of toluene and d^8-toluene was used as the substrate for the measurement of kinetic isotope effects (KIE). The KIE value was calculated by dividing the mole of electrons required for the formation of normal products by the corresponding mole of electrons for deuterated products.

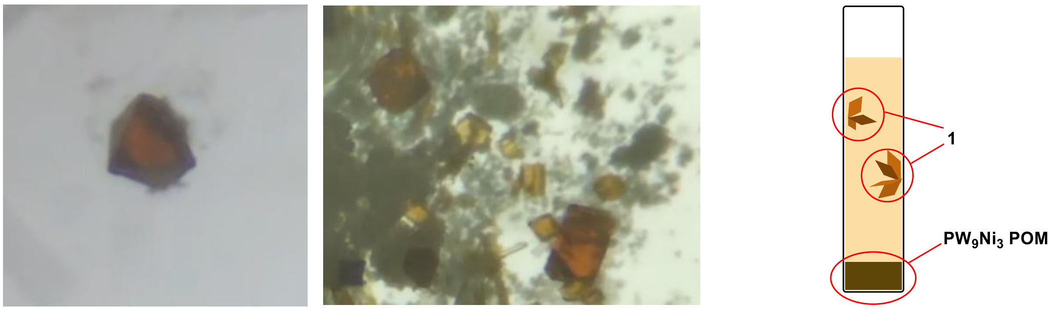

Figure S1. Typical crystallites of **1** with different size (left) and the illustration on the separation of **1** from $Cs_4K[PW_9Ni^{III}{}_2Ni^{II}O_{40}H_8]\cdot 6H_2O$ (right) in sample vials used for crystallization.

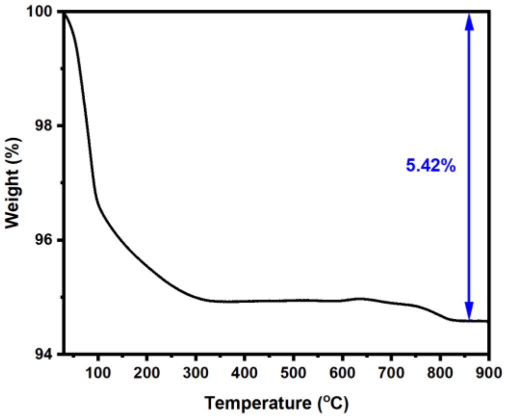

Figure S2. Thermogravimetric curve of **1**.

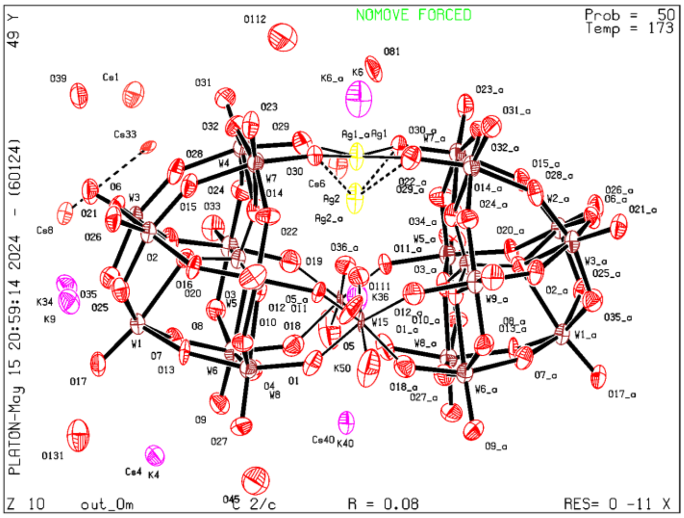

Figure S3. Schematic illustration of the crystal structure of **1** showing thermal ellipsoids.

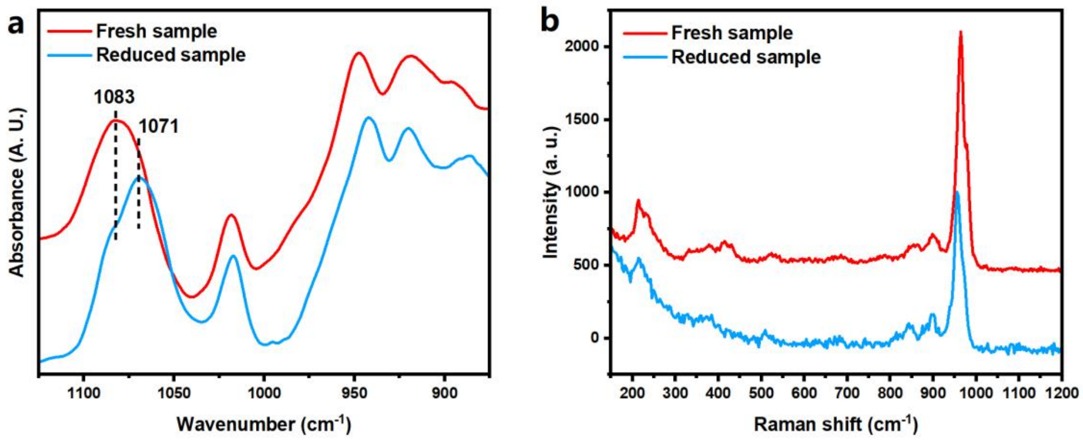

Figure S4. Comparison of (a) IR spectra and (b) Raman spectra of freshly-prepared and partially reduced samples of **1**.

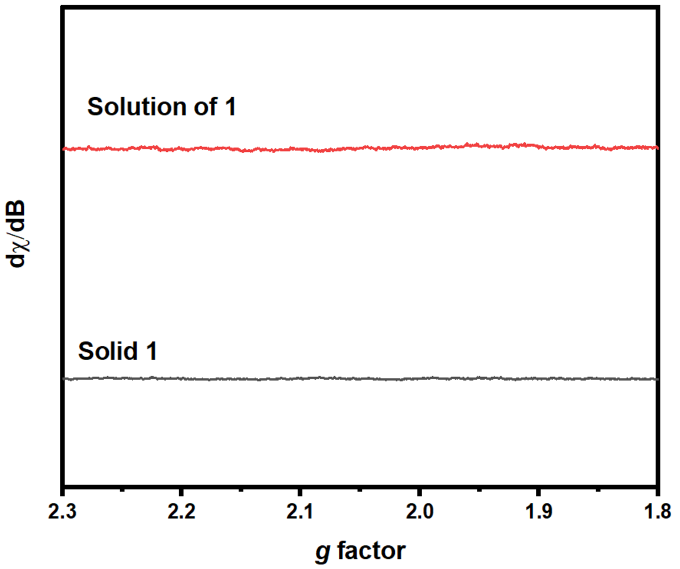

Figure S5. EPR spectrum of solution and solid samples of **1** under 300K.

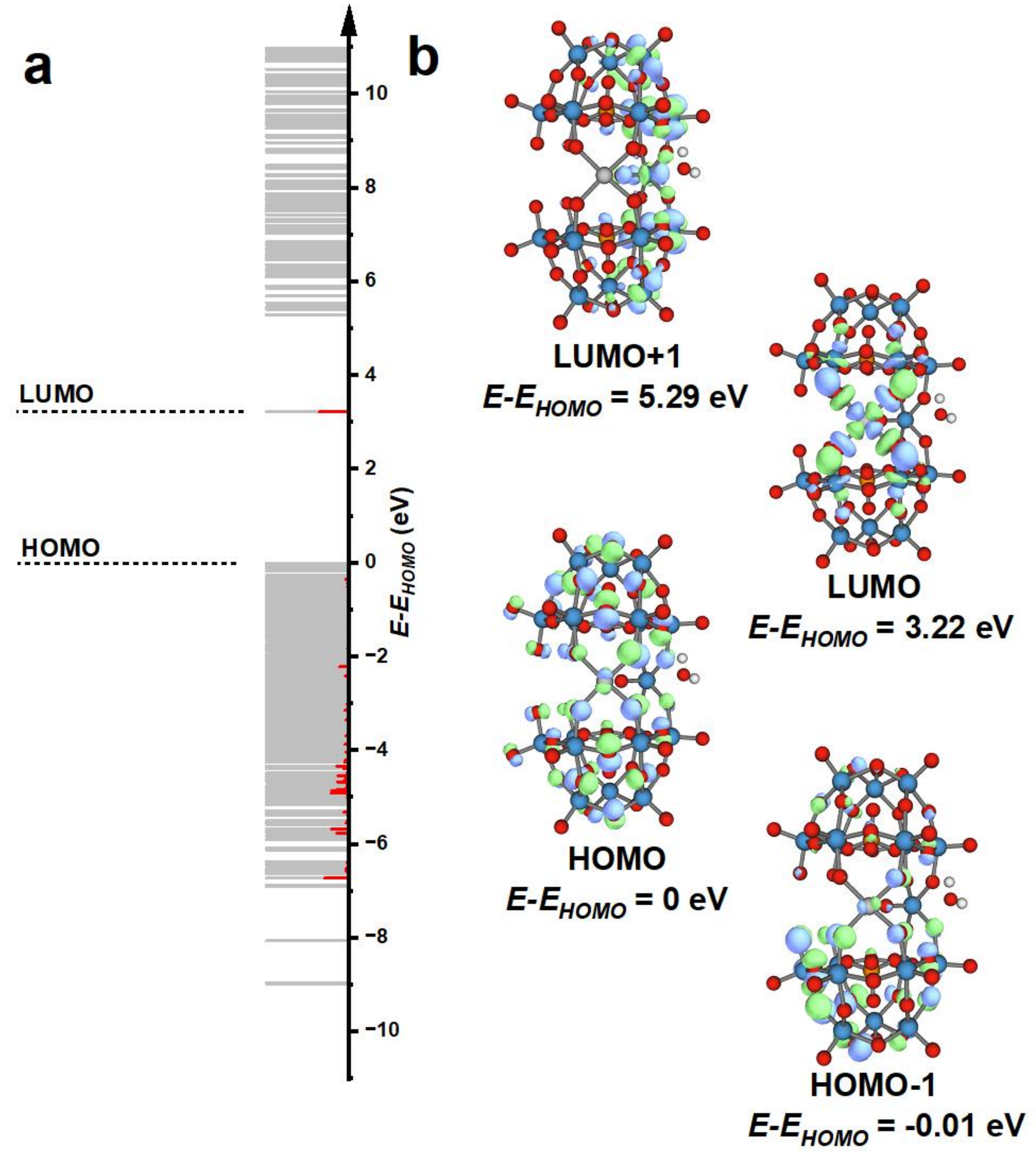

Figure S6. Molecular orbital (MO) analysis of $[P_2W_{19}Ag^{III}O_{70}H_2]^{11-}$. (**a**) The MO energy level diagram of $[P_2W_{19}Ag^{III}O_{70}H_2]^{11-}$, with MOs represented as light grey bars. The contributions from Ag 4d orbital components to each molecular orbital are illustrated as the relative length of the red bars. (**b**) Visualized distribution of frontier molecular orbitals of $[P_2W_{19}Ag^{III}O_{70}H_2]^{11-}$.

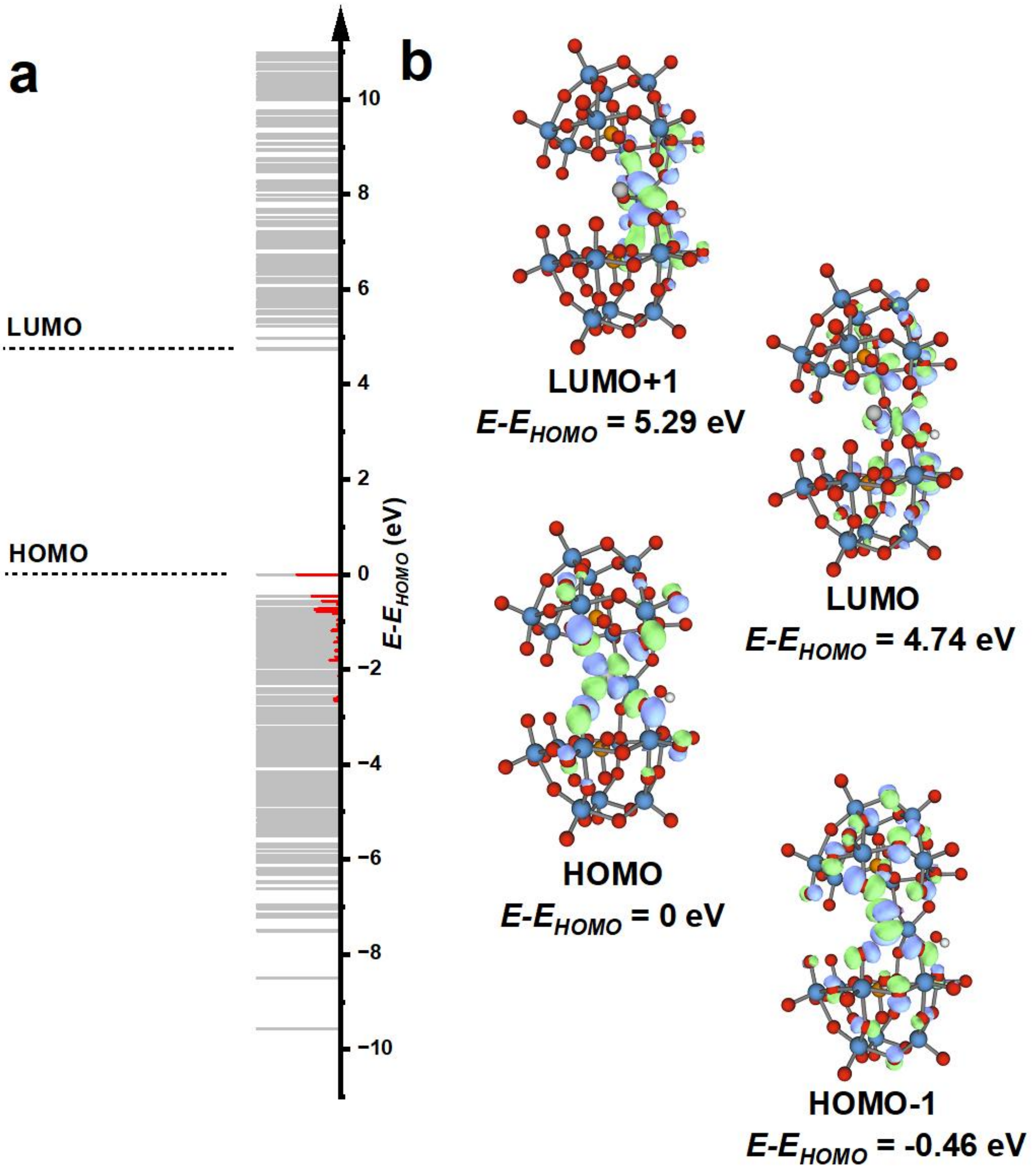

Figure S7. Molecular orbital (MO) analysis of $[P_2W_{19}Ag^IO_{70}H_2]^{13-}$. (**a**) The MO energy level diagram of $[P_2W_{19}Ag^IO_{70}H_2]^{13-}$, with MOs represented as light grey bars. The contributions from Ag 4d orbital components to each molecular orbital are illustrated as the relative length of the red bars. (**b**) Visualized distribution of frontier molecular orbitals of $[P_2W_{19}Ag^IO_{70}H_2]^{13-}$.

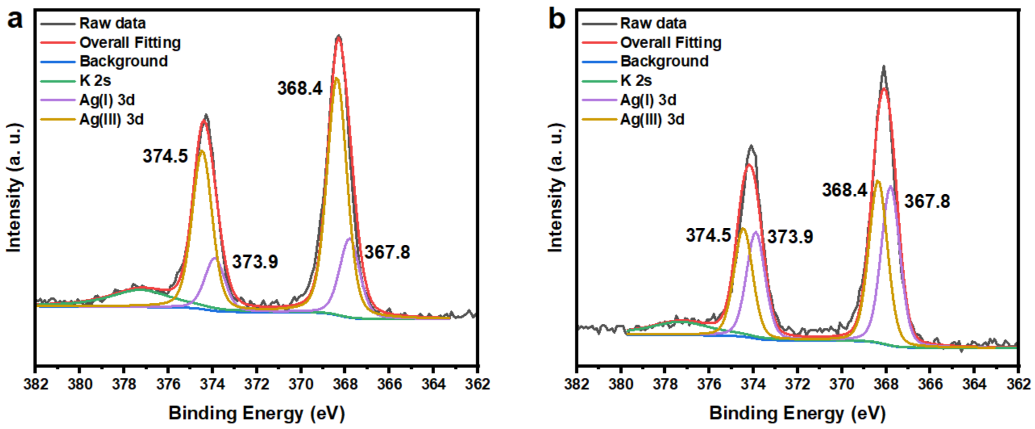

Figure S8. X-ray photoelectron spectra of (**a**) fresh sample and (**b**) partially reduced sample of **1**. Peak positions are labelled in the figures.

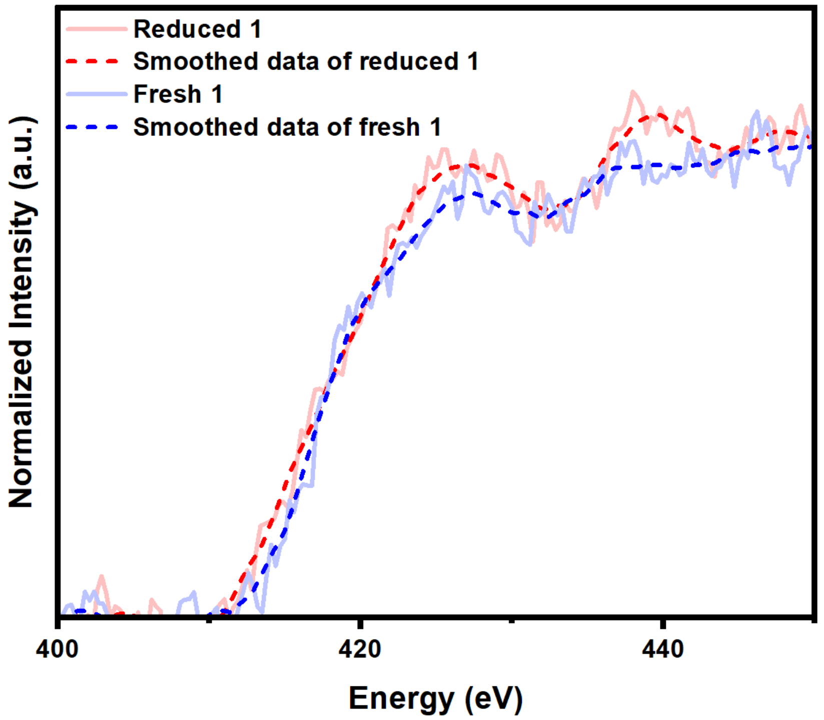

Figure S9. Electron energy loss spectra of fresh sample (blue) and reduced sample (red) of **1**. Corresponding 15 points adjacent-averaging smoothed data are shown to highlight the edge profile.

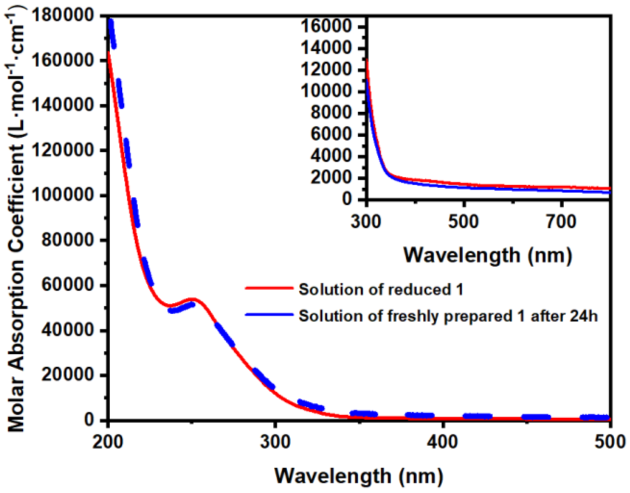

Figure S10. UV-Vis spectra of reduced sample of **1** in aqueous solution (red line) and solution of fresh sample of **1** after 24 hours (blue line).

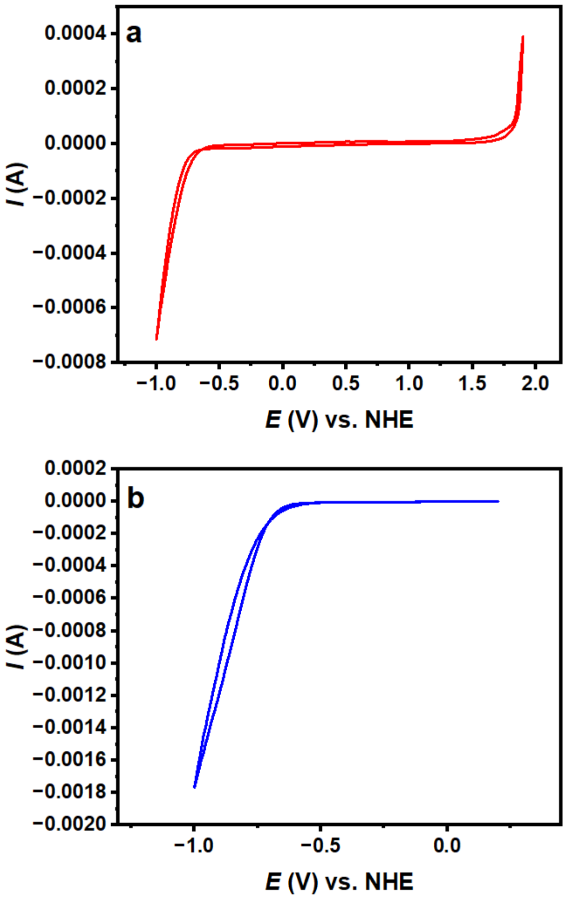

Figure S11. Cyclic voltammogram of (**a**) overall scan of **1** in the range of -1.0~1.9 V vs. NHE and (**b**) detailed scan in the range of -1.0 ~ 0.2 V vs. NHE under a scan rate of 5 mV/s.

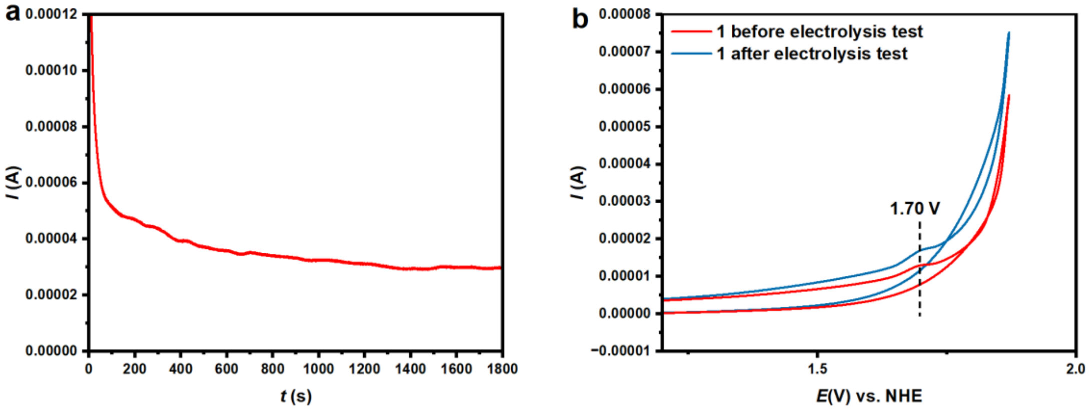

Figure S12. (a) Chronoamperometric curve of **1** in 0.5 M $NaNO_3$ solution under 1.85 V vs. NHE for 1800 s using a glassy carbon electrode (S=0.07cm^2) as the working electrode. (b) Cyclic voltammogram of **1** before and after the corresponding electrolysis test. The increase in current after electrolysis test under 1.85 V vs. NHE may be attributed to the change in surface status of the glassy carbon electrode due to oxidation.

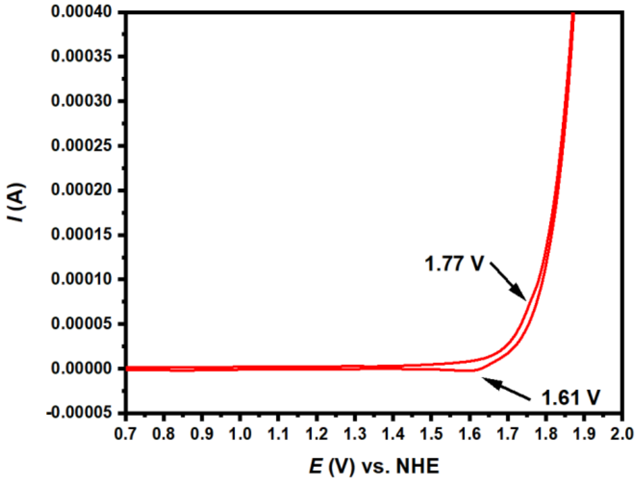

Figure S13. Cyclic voltammogram of $AgNO_3$ in the range of 0.7~2.0 V vs. NHE. Working electrode: glassy carbon; reference: Ag/AgCl (saturated); Scan rate: 5 mV/s.

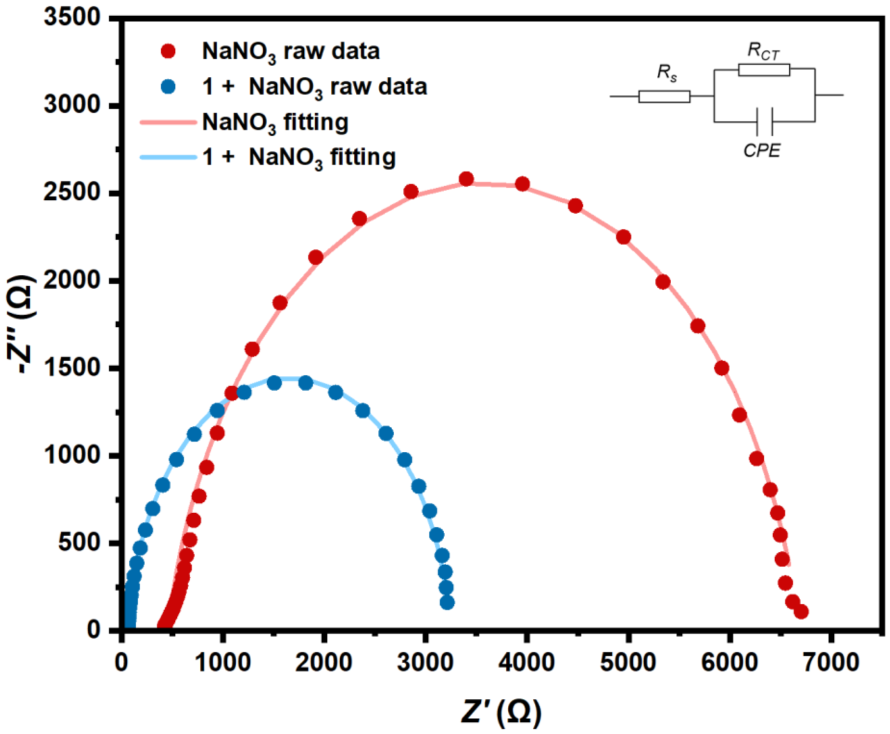

Figure S14. Electrochemical impedance spectra of the glassy carbon electrode in 0.5 M $NaNO_3$ with (blue) and without (red) **1**. Fitting results corresponding to the equivalent circuit shown in the figure are shown as lines.

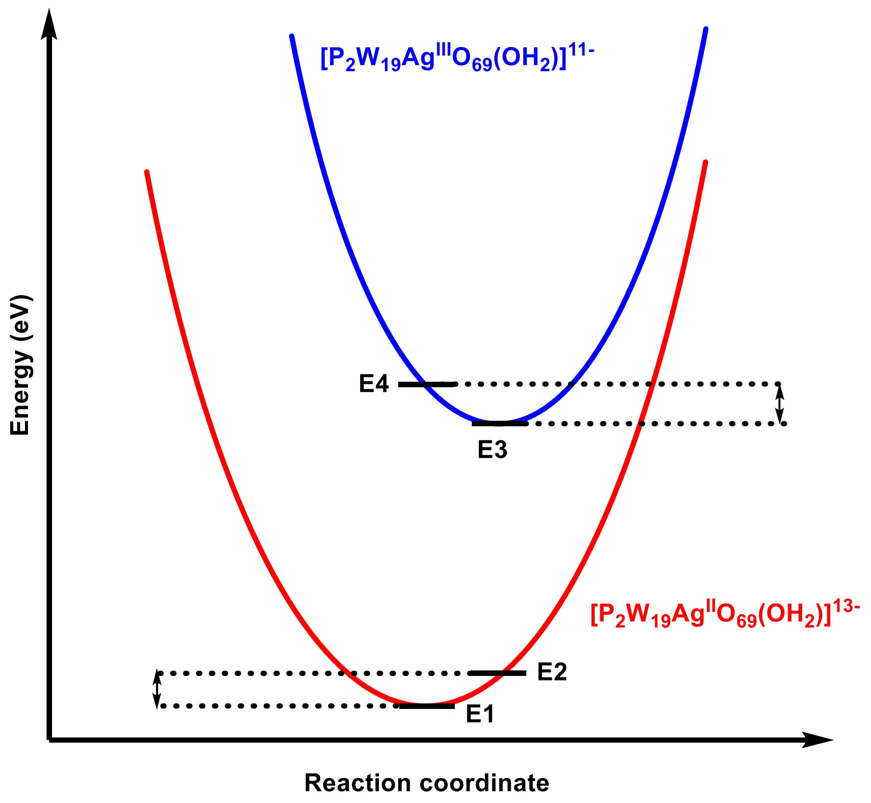

Figure S15. Schematic illustration of the calculation of Marcus internal reorganization energy (λ_i) based on Nelson's method, taking $[P_2W_{19}Ag^{III}O_{70}H_2]^{11-}$/$[P_2W_{19}Ag^{II}O_{70}H_2]^{12-}$ redox pair as an example. In this figure, E1 and E3 represent the energy of $[P_2W_{19}Ag^{II}O_{70}H_2]^{12-}$ and $[P_2W_{19}Ag^{III}O_{70}H_2]^{11-}$ under their optimised structures, E2 the energy of $[P_2W_{19}Ag^{II}O_{70}H_2]^{11-}$ with the same geometry as the optimised structure of $[P_2W_{19}Ag^{III}O_{70}H_2]^{12-}$, and E4 the energy of $[P_2W_{19}Ag^{III}O_{70}H_2]^{12-}$ with the same geometry as the optimised structure of $[P_2W_{19}Ag^{II}O_{70}H_2]^{11-}$. The overall λ_i value of the self-exchange reaction can be calculated based on the formula λ_i=|E2-E1|+|E4-E3|.

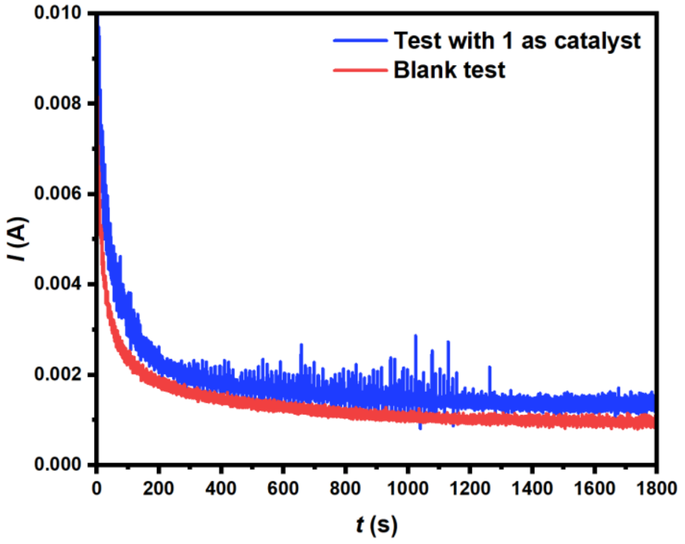

Figure S16. Typical chronoamperometric curve of the electrochemical oxidation of toluene with (blue) and without (red) the presence of **1**, corresponding to the condition b and a in **Table 1**, respectively.

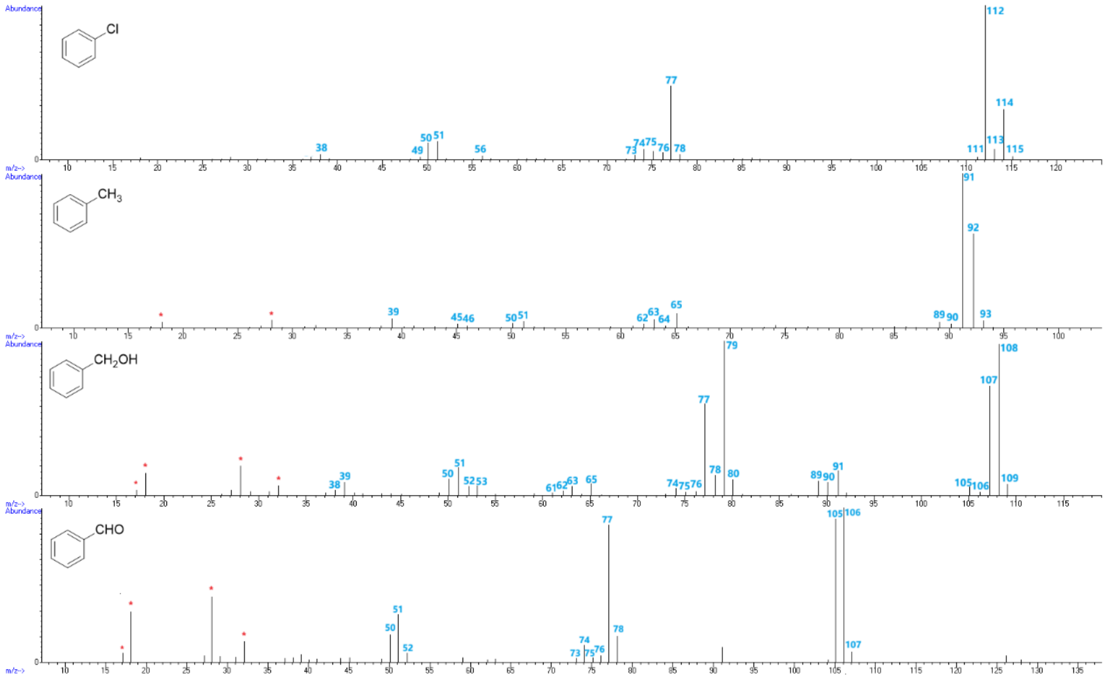

Figure S17. GC-MS mass spectra of chlorobenzene (internal standard) and the substances occurring in the electrochemical oxidation of toluene, with the *m/z* value of peaks labelled in blue. Background peaks from the small amount of air and water are marked with asterisks. Note that in the mass spectra of some products these are relatively more significant due to the low concentration of the substrate.

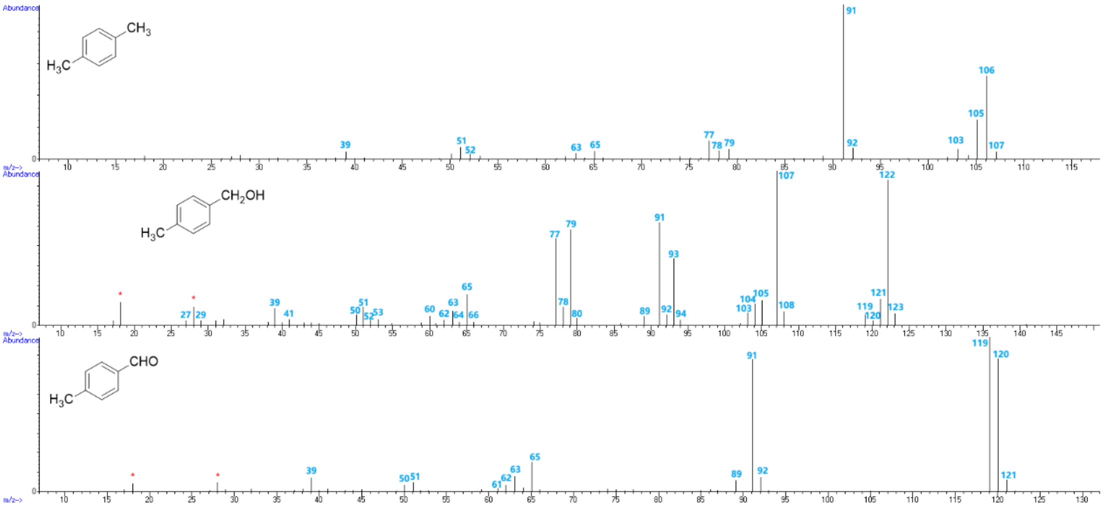

Figure S18. GC-MS mass spectra of the substances occurring in the electrochemical oxidation of xylene, with the *m/z* value of peaks labelled in blue. Background peaks from the small amount of air and water are marked with asterisks.

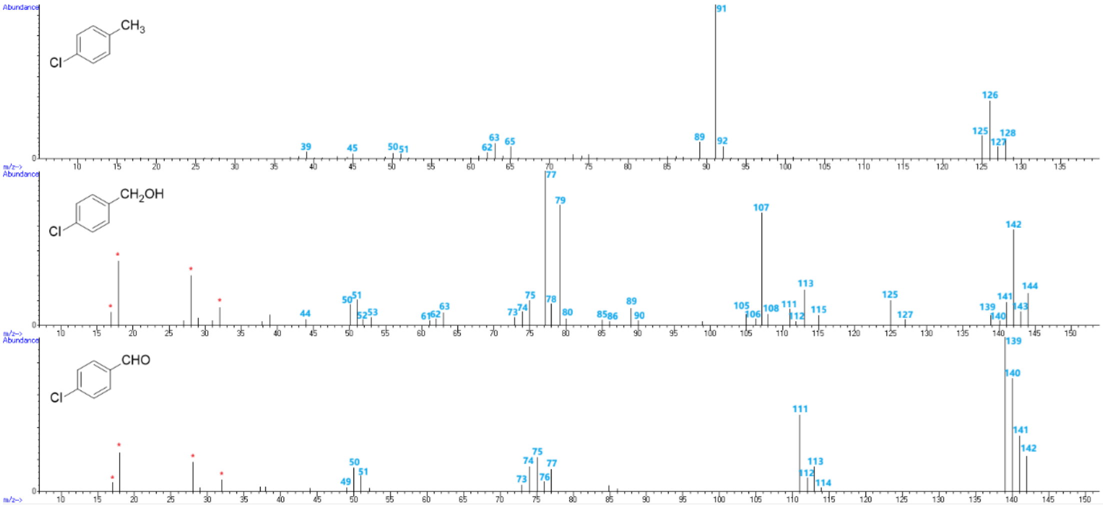

Figure S19. GC-MS mass spectra of the substances occurring in the electrochemical oxidation of 4-chlorotoluene, with the *m/z* value of peaks labelled in blue. Background peaks from the small amount of air and water are marked with asterisks.

Table S1. Crystal data of **1**.

**Compound**	**1**
Empirical formula	$Cs_7K_4P_2W_{19}AgO_{83}H_{28}$
Formula weight	6105.68
Temperature (K)	173(2)
Wavelength (Å)	1.34138
Crystal system	Monoclinic
Space group	*C2/c*
a (Å)	20.5360(16)
b (Å)	16.0951(13)
c (Å)	26.627(2)
α (°)	90
β (°)	102.454(3)
γ (°)	90
Volume (Å^3)	8593.9(12)
Z	8
F(000)	10325
θ range (°)	3.063 to 57.151
Reflections collected	60872
GooF on F^2	0.960
R_1 $[I > 2\sigma(I)]^a$	0.0849
wR_2^b	0.2487

$^aR_1 = \sum||F_o|-|F_c||/\sum|F_o|$.

$^b wR_2 = [\sum w(F_o^2-F_c^2)^2/\sum w(F_o^2)^2]^{1/2}$.

Table S2. BVS calculation of selected atoms in **1**.

**Atom 1**	**Atom 2**	***R_0* value**[22]	***B* value**[22]	**Length (Å)**	**Bond valence**	**Valence sum**
Ag1	O29	1.91	0.370	2.035	0.713	2.823
	O30	1.91	0.370	2.043	0.698	
Ag2	O29	1.805	0.370	2.483	0.160	0.769
	O30	1.805	0.370	2.471	0.165	
	O36	1.805	0.370	2.594	0.119	

Table S3. Cartesian structural coordinates of optimized structure of $[P_2W_{19}Ag^{III}O_{70}H_2]^{11-}$.

**Atom**	**x**	**y**	**z**
Ag	0.031165	2.564896	-1.80325
O	-3.34615	1.764844	-4.82123
O	-3.53921	5.195111	-0.49272
O	5.177931	2.855418	-0.43712
O	4.922954	0.766201	-2.75218
O	2.709076	-0.54262	-3.11744
O	3.201454	3.20464	1.234988
O	3.396734	-3.68566	-1.20641
O	3.927795	0.785102	4.002209
O	1.243332	-2.52815	-2.08342
O	1.761928	2.32516	3.318004
O	1.337054	-3.34694	0.415454
O	1.674899	-0.76448	3.883505
O	-4.89027	-1.65254	-2.35483
O	-5.33637	1.976995	2.094848
O	5.095548	-2.83257	0.675759

Atom	x	y	z
O	5.279121	-1.10953	2.611213
O	3.446489	-4.98161	1.242887
O	3.909948	-1.85795	4.927857
O	3.038545	-2.43828	2.27961
O	7.676948	2.975339	0.846206
O	7.295942	-0.66679	-3.30607
O	7.608484	-2.46238	1.928031
O	2.940928	-1.31154	-0.3299
O	3.025284	0.363781	1.607139
O	2.900594	1.190347	-0.79966
O	3.186241	-3.17785	-3.94764
O	4.066803	3.584673	3.926258
O	-1.25428	-2.35663	-2.10166
O	-1.72078	2.160665	3.39542
O	1.320866	1.713514	-3.02998
O	1.479413	3.559954	-0.8435
O	-0.34333	-4.8196	-1.60225
O	-2.7296	-0.42842	-3.16666
O	-3.11425	3.147428	1.323619
O	-2.8877	1.215783	-0.76687
O	-4.91362	0.891178	-2.74032
O	-5.10984	2.909684	-0.35452
O	-7.28621	-0.50943	-3.34251
O	-7.61636	3.032181	0.908049
O	0.024879	-0.92491	-0.04935

Atom	x	y	z
O	-1.30531	1.78835	-3.02313
O	-1.38475	3.533776	-0.75237
O	-3.49929	3.153799	-2.39376
O	-3.52434	-5.00508	1.079666
O	-4.01899	-1.98332	4.891181
O	-3.37064	-3.64499	-1.31561
O	-3.93572	0.675784	4.024867
O	-2.97271	-1.29358	-0.36042
O	-3.02911	0.311011	1.62294
O	-3.15455	-3.04867	-4.01714
O	-3.9918	3.480788	4.015562
O	5.012866	0.045147	0.029686
O	7.047532	1.090738	-1.1744
O	-5.14898	-2.81607	0.561516
O	-5.32103	-1.15993	2.564191
O	-7.01906	1.188501	-1.15623
O	-6.97703	-1.445	-0.73243
O	-7.24518	0.261564	1.280884
O	-7.66985	-2.44154	1.824496
O	-3.13734	-2.52315	2.234055
O	3.558382	3.059277	-2.48277
O	-5.01812	0.08439	0.026913
P	3.431399	0.078885	0.141805
P	-3.43693	0.090417	0.147426
W	3.089176	-2.28155	-2.48505

Atom	x	y	z
W	3.450542	2.305087	2.939162
W	6.387354	-1.46664	1.235609
W	3.297345	-3.36526	0.680077
W	3.354882	-0.98566	3.540992
W	6.396665	1.853109	0.574242
W	6.239294	-0.3825	-1.98136
W	-3.08415	-2.17305	-2.53905
W	-3.40507	2.20902	3.000782
W	-3.36715	-3.36554	0.588911
W	-3.41865	-1.09913	3.531865
W	-3.12243	1.451651	-3.14536
W	-3.22011	3.504255	-0.50712
W	-6.23274	-0.27487	-2.00591
W	-6.35106	1.899218	0.609465
W	-6.43225	-1.43885	1.173227
W	3.132765	1.33621	-3.15768
W	3.31607	3.491106	-0.60708
W	-0.00781	-2.53369	-0.61143
O	3.342577	1.584476	-4.84664
O	3.670011	5.173914	-0.65863
O	7.243325	0.22482	1.288181
O	6.950908	-1.52808	-0.66879
O	-1.3931	-3.30876	0.379859
O	-1.73191	-0.96642	3.890533
O	4.886559	-1.75166	-2.30858

Atom	x	y	z
O	5.372031	2.000876	2.046327
H	-0.11814	-4.57512	-2.51432
H	-1.31513	-4.7397	-1.56726

Table S4. Cartesian structural coordinates of optimized structure of $[P_2W_{19}Ag^{II}O_{70}H_2]^{12-}$.

Atom	x	y	z
Ag	-1.00497	2.957472	-0.03659
O	-5.68248	2.438867	-0.67483
O	-3.25791	3.828173	4.105826
O	3.998036	4.125857	-1.9005
O	2.334467	3.015954	-4.30398
O	0.318838	1.436853	-3.97007
O	3.459064	3.35745	0.525176
O	1.917668	-1.91403	-4.36184
O	5.581326	0.316186	1.030993
O	-0.29795	-1.07419	-3.2878
O	3.464152	1.429282	2.390613
O	1.267956	-2.70185	-1.93688
O	3.685386	-1.60729	1.473706
O	-5.34962	-1.99504	0.232421
O	-3.01442	-0.56652	5.323803
O	4.428389	-1.41313	-3.57858
O	5.772109	-0.53789	-1.54869
O	3.406621	-3.96052	-3.2355
O	6.083906	-2.38542	0.512354

Atom	x	y	z
O	3.772341	-2.17898	-1.14013
O	6.760214	4.378106	-2.29298
O	3.838752	2.558946	-6.63865
O	7.200744	-0.91489	-3.91145
O	2.150291	-0.20683	-2.43832
O	3.423424	0.599458	-0.36551
O	1.894665	2.195707	-1.62332
O	0.123882	-0.50356	-5.97405
O	5.73317	2.873106	2.151406
O	-2.30575	-1.58333	-1.85512
O	0.60604	0.215932	4.334987
O	-0.70988	3.041188	-2.13181
O	0.843722	4.006406	0.220427
O	-1.38401	-3.7265	-3.13635
O	-4.17033	0.047279	-0.91039
O	-1.78021	1.454363	4.217079
O	-2.76887	0.554868	1.62485
O	-5.60607	0.391429	1.190561
O	-4.36428	1.246427	3.955533
O	-7.85012	-1.28403	1.36618
O	-5.55791	0.120756	6.2204
O	-0.03778	-0.72102	-0.45311
O	-2.96723	2.378742	-0.54897
O	-1.63461	3.086504	2.037341
O	-4.30049	2.681283	1.761567

Atom	x	y	z
O	-2.14259	-5.79239	0.312609
O	-0.23249	-4.68015	4.692472
O	-3.47333	-3.65797	-0.81768
O	-0.72005	-1.99425	5.293863
O	-2.62345	-1.83493	0.778868
O	-1.46142	-1.17992	2.958309
O	-4.97571	-2.07107	-2.51939
O	-0.76488	0.435557	6.657838
O	4.05472	1.396123	-2.71114
O	5.005902	3.263568	-4.22461
O	-3.76059	-4.15288	1.851264
O	-2.69155	-3.49646	4.124943
O	-6.33176	-0.55972	3.580083
O	-5.99893	-3.00271	2.578572
O	-5.00135	-2.34303	4.954876
O	-5.00076	-5.04024	4.259094
O	-1.14211	-3.92956	2.095084
O	1.449329	4.643808	-2.34888
O	-4.00825	-1.34755	2.798969
P	2.858156	0.989162	-1.75382
P	-2.68004	-0.93041	2.034701
W	0.956717	-0.25244	-4.48869
W	4.607937	1.962998	1.199635
W	5.840894	-0.05299	-3.29339
W	2.993609	-2.33906	-2.83167

Atom	x	y	z
W	4.817982	-1.24109	0.214871
W	5.526209	3.167066	-2.27624
W	3.79404	2.067465	-4.98827
W	-4.03564	-1.82088	-1.09784
W	-0.95429	-0.10401	5.023387
W	-2.33095	-4.11816	0.670303
W	-0.60608	-3.22329	3.832552
W	-4.42619	1.62292	0.178947
W	-2.92794	2.456017	3.114701
W	-6.19762	-1.25362	1.850116
W	-4.72472	-0.35591	4.782557
W	-4.4326	-3.53818	3.631262
W	0.615653	3.241339	-3.33112
W	2.374139	4.310221	-0.68037
W	-0.41686	-1.948	-1.57787
O	-0.18066	4.155015	-4.55936
O	2.709516	5.939428	-0.22106
O	6.608491	1.629763	-2.95797
O	5.133578	0.710788	-4.9601
O	-0.89392	-3.40315	-0.49906
O	0.947516	-2.73251	3.246228
O	2.512459	0.580962	-5.1185
O	5.624011	2.477096	-0.61352
H	-1.77336	-3.07823	-3.74449
H	-2.09771	-3.89565	-2.48846

Table S5. Cartesian structural coordinates of optimized structure of $[P_2W_{19}Ag^IO_{70}H_2]^{13-}$.

Atom	x	y	z
P	-2.59244	2.200477	1.061705
P	2.714393	-1.95193	-1.24347
O	-3.79352	3.046753	1.654214
O	3.832341	-2.97559	-1.70535
O	-1.37095	3.1312	0.865901
O	2.188914	-2.47724	0.116117
O	-3.0106	1.579065	-0.291
O	1.580093	-1.93929	-2.29143
O	-2.30632	1.089003	2.102869
O	3.337159	-0.54121	-1.10213
W	-5.92872	2.142988	2.200734
W	5.010541	-2.77002	-3.71382
W	-3.68401	4.198739	3.738346
W	5.807047	-3.42921	-0.47026
W	-4.74391	4.891694	0.577666
W	3.448678	-5.30452	-2.05758
W	-0.04418	4.247694	2.471218
W	3.323227	-2.60738	2.129597
W	-1.15905	5.016838	-0.60715
W	1.002335	-4.47077	0.576913
W	-3.61803	2.74541	-2.30084
W	0.08865	-3.71394	-2.93565
W	-4.75821	-0.02413	-0.72336
W	1.678696	-1.21044	-4.5576

**Atom**	**x**	**y**	**z**
W	-1.4545	1.307367	4.251705
W	4.992958	0.227863	0.414569
W	-3.6671	-0.72189	2.749554
W	4.304664	0.831879	-2.82299
O	-5.29904	3.105611	3.748436
O	-2.00156	4.637503	3.201177
O	4.599772	-3.5083	1.134179
O	-4.53806	5.348188	2.539479
O	4.977754	-5.13969	-0.89311
O	-0.46344	5.533365	1.115912
O	2.150663	-4.13478	2.088176
O	-3.04435	5.446451	0.316965
O	2.419202	-5.24137	-0.35739
O	-4.76946	3.828983	-0.89912
O	1.884346	-4.73081	-2.84047
O	-2.33287	4.032991	-1.75526
O	0.118691	-4.19574	-1.04535
O	-5.71287	1.218715	0.62484
O	3.393003	-2.47335	-4.48343
O	-2.82858	2.538005	4.459131
O	5.810399	-1.63219	-0.17184
O	-4.92524	0.621568	3.009182
O	5.248517	-1.04593	-3.22734
O	-4.17943	-0.83454	0.95399
O	2.978662	0.03342	-3.94723

Atom	x	y	z
O	-4.966	1.393486	-1.99066
O	0.749074	-2.90986	-4.54022
O	-6.25041	3.769438	1.347852
O	4.504925	-4.72049	-3.48154
O	-2.70372	-0.14838	4.316006
O	5.513302	0.840946	-1.3195
O	-0.3622	2.643998	3.536103
O	4.096871	-0.97647	1.663757
O	-2.4392	1.661123	-3.02515
O	-1.15206	-2.51518	-2.63607
O	-0.24014	-0.01305	3.413325
O	3.901593	1.522969	0.789784
O	-1.99241	-1.59447	2.260665
O	3.27081	2.116077	-2.26519
O	0.691263	5.287793	3.649076
O	3.928096	-2.81535	3.732266
O	-3.88243	5.062561	5.220722
O	7.271386	-3.97124	0.268899
O	-0.8531	1.210333	5.864548
O	6.409159	0.656059	1.320374
O	-5.69684	6.261411	0.109203
O	3.433935	-7.00954	-2.32927
O	-3.49582	-0.86487	-1.59636
O	0.296929	-0.23467	-4.0927
O	-4.49354	-2.07575	3.423872

Atom	x	y	z
O	5.299332	1.643755	-3.98801
O	-0.11911	-3.02198	4.06539
O	-7.52264	1.713672	2.706662
O	-1.1272	6.560245	-1.39634
O	0.197325	-5.87576	1.169237
O	-0.01392	-0.36201	0.623485
O	1.323496	3.540765	1.672756
O	1.668151	-1.61464	2.582138
O	0.294911	4.266533	-1.19909
O	-0.13588	-3.10931	1.368518
Ag	-1.1678	-0.17842	-2.00917
W	-0.12911	-1.30589	2.054748
O	6.075585	-2.96613	-5.06752
O	6.326707	-3.29671	-2.25397
O	-4.32042	3.522924	-3.67988
O	-0.80844	-5.0363	-3.60388
O	-6.16491	-0.96739	-1.08508
O	1.766372	-0.97181	-6.2716
H	-0.34581	-3.65226	3.358746
H	0.847151	-2.94789	3.967562

Table S6. Calculated relative energies used for the Marcus internal reorganization energy (λi) calculation based on Nelson's four-points method, comparing to the E1 of each redox pair (all relative E1 value are set to 0 kcal/mol).

Redox Pair	E2 (kcal/mol)	E3 (kcal/mol)	E4 (kcal/mol)
$[P_2W_{19}Ag^{III}O_{70}H_2]^{11-}$ /$[P_2W_{19}Ag^{II}O_{70}H_2]^{12-}$	18.2	115.8	136.4
$[P_2W_{19}Ag^{II}O_{70}H_2]^{12-}$ /$[P_2W_{19}Ag^{I}O_{70}H_2]^{13-}$	18.3	100.6	133.5

Table S7. Calculated Marcus internal reorganization energy (λ_i) and activation energy E_a of the $[P_2W_{19}Ag^{III}O_{70}H_2]^{11-}$/$[P_2W_{19}Ag^{II}O_{70}H_2]^{12-}$ and $[P_2W_{19}Ag^{II}O_{70}H_2]^{12-}$/$[P_2W_{19}Ag^{I}O_{70}H_2]^{13-}$ redox pairs based on Nelson's method.

Redox Pair	λ_i (kcal/mol)[a]	E_a(kcal/mol)[b]
$[P_2W_{19}Ag^{III}O_{70}H_2]^{11-}$ /$[P_2W_{19}Ag^{II}O_{70}H_2]^{12-}$	38.7	14.2
$[P_2W_{19}Ag^{II}O_{70}H_2]^{12-}$ /$[P_2W_{19}Ag^{I}O_{70}H_2]^{13-}$	51.3	17.3

[a] Calculated under PBE0-D3(BJ)/def2tzvp level based on $[P_2W_{19}AgO_{70}H_2]^{x-}$ with Ag centres of corresponding oxidation state.

[b] Calculated based on the formula $E_a = \frac{(\lambda+\Delta G°)^2}{4\lambda}$, where $\Delta G° = 0$ for self-exchange reactions, and $\lambda = \lambda_i + \lambda_e$ with λ_e set to 18 kcal/mol[17,18].

References

(1) Finke, R. G.; Droege, M. W.; Domaille, P. J. Trivacant Heteropolytungstate Derivatives. 3. Rational Syntheses, Characterization, Two-Dimensional Tungsten-183 NMR, and Properties of Tungstometallophosphates $P_2W_{18}M_4(H_2O)_2O_{68}^{10-}$ and $P_4W_{30}M_4(H_2O)_2O_{112}^{16-}$ (M = Cobalt, Copper, Zinc). *Inorganic Chemistry* **1987**, *26* (23), 3886–3896. https://doi.org/10.1021/ic00270a014.

(2) Frisch, M. J.; Trucks, G. W.; Schlegel, H. B.; Scuseria, G. E.; Robb, M. A.; Cheeseman, J. R.; Scalmani, G.; Barone, V.; Petersson, G. A.; Nakatsuji, H., et al. Gaussian 16, revision A.03; Gaussian, Inc.: Wallingford, CT, **2016**.

(3) Richards, A. *University of Oxford Advanced Research Computing*. Zenodo. University of Oxford Advanced Research Computing.

(4) Grimme, S.; Antony, J.; Ehrlich, S.; Krieg, H. A Consistent and Accurate Ab Initio Parametrization of Density Functional Dispersion Correction (DFT-D) for the 94 Elements H-Pu. *The Journal of Chemical Physics* **2010**, *132* (15), 154104. https://doi.org/10.1063/1.3382344.

(5) Johnson, E. R.; Becke, A. D. A Post-Hartree-Fock Model of Intermolecular Interactions: Inclusion of Higher-Order Corrections. *The Journal of Chemical Physics* **2006**, *124* (17), 174104–174104. https://doi.org/10.1063/1.2190220.

(6) Ditchfield, R.; Hehre, W. J.; Pople, J. A. Self-Consistent Molecular-Orbital Methods. IX. An Extended Gaussian-Type Basis for Molecular-Orbital Studies of Organic Molecules. *The Journal of Chemical Physics* **1971**, *54* (2), 724–728. https://doi.org/10.1063/1.1674902.

(7) Hariharan, P. C.; Pople, J. A. The Influence of Polarization Functions on Molecular Orbital Hydrogenation Energies. *Theoretica Chimica Acta* **1973**, *28* (3), 213–222. https://doi.org/10.1007/bf00533485.

(8) Hehre, W. J.; Ditchfield, R.; Pople, J. A. Self—Consistent Molecular Orbital Methods. XII. Further Extensions of Gaussian—Type Basis Sets for Use in Molecular Orbital Studies of Organic Molecules. *The Journal of Chemical Physics* **1972**, *56* (5), 2257–2261. https://doi.org/10.1063/1.1677527.

(9) Francl, M. M.; Pietro, W. J.; Hehre, W. J.; Binkley, J. S.; Gordon, M. S.; DeFrees, D. J.; Pople, J. A. Self-Consistent Molecular Orbital Methods. XXIII. A Polarization-Type Basis Set for Second-Row Elements. *The Journal of Chemical Physics* **1982**, *77* (7), 3654–3665. https://doi.org/10.1063/1.444267.

(10) Hay, P. J.; Wadt, W. R. Ab Initio Effective Core Potentials for Molecular Calculations. Potentials for K to Au Including the Outermost Core Orbitals. *The Journal of Chemical Physics* **1985**, *82* (1), 299–310. https://doi.org/10.1063/1.448975.

(11) Weigend, F.; Ahlrichs, R. Balanced Basis Sets of Split Valence, Triple Zeta Valence and Quadruple Zeta Valence Quality for H to Rn: Design and Assessment of Accuracy. *Physical Chemistry Chemical Physics* **2005**, *7* (18), 3297. https://doi.org/10.1039/b508541a.

(12) Liu, Z.; Lu, T.; Chen, Q. An Sp-Hybridized All-Carboatomic Ring, Cyclo[18]Carbon: Electronic Structure, Electronic Spectrum, and Optical Nonlinearity. *Carbon* **2020**, *165*, 461–467. https://doi.org/10.1016/j.carbon.2020.05.023.

(13) Alex; Sundstrom, E.; Head-Gordon, M. LOBA: A Localized Orbital Bonding Analysis to Calculate Oxidation States, with Application to a Model Water Oxidation Catalyst. *Physical Chemistry Chemical Physics* **2009**, *11* (47), 11297–11297. https://doi.org/10.1039/b915364k.

(14) Lu, T.; Chen, F. Multiwfn: A Multifunctional Wavefunction Analyzer. *Journal of Computational Chemistry* **2011**, *33* (5), 580–592. https://doi.org/10.1002/jcc.22885.

(15) Marcus, R. A. On the Theory of Oxidation-Reduction Reactions Involving Electron Transfer. I. *The Journal of Chemical Physics* **1956**, *24* (5), 966–978. https://doi.org/10.1063/1.1742723.

(16) Nelsen, S. F.; Blackstock, S. C.; Kim, Y. Estimation of Inner Shell Marcus Terms for Amino Nitrogen Compounds by Molecular Orbital Calculations. *Journal of the American Chemical Society* **1987**, *109* (3), 677–682. https://doi.org/10.1021/ja00237a007.
(17) Weinstock, I. A.; Schreiber, R. E.; Neumann, R. Dioxygen in Polyoxometalate Mediated Reactions. *Chemical Reviews* **2017**, *118* (5), 2680–2717. https://doi.org/10.1021/acs.chemrev.7b00444.
(18) Weinstock, I. A. Homogeneous-Phase Electron-Transfer Reactions of Polyoxometalates. *Chemical Reviews* **1998**, *98* (1), 113–170. https://doi.org/10.1021/cr9703414.
(19) Sheldrick, G. M. Crystal Structure Refinement with SHELXL. *Acta Crystallographica Section C Structural Chemistry* **2015**, *71* (1), 3–8. https://doi.org/10.1107/s2053229614024218.
(20) Momma, K.; Izumi, F. VESTA 3 for Three-Dimensional Visualization of Crystal, Volumetric and Morphology Data. *Journal of Applied Crystallography* **2011**, *44* (6), 1272–1276. https://doi.org/10.1107/s0021889811038970.
(21) Pomerantsev, A. L. *Progress in Chemometrics Research*; Nova Science Publishers: New York, 2005; pp. 89–102.
(22) Brese, N. E.; O'Keeffe, M. Bond-Valence Parameters for Solids. *Acta Crystallographica Section B Structural Science* **1991**, *47* (2), 192–197. https://doi.org/10.1107/s0108768190011041.
